# Ossification patterns of the carpus and tarsus in salamanders and impacts of preaxial dominance on the fin-to-limb transition

**DOI:** 10.1126/sciadv.abq7669

**Published:** 2022-10-14

**Authors:** Jia Jia, Jason S. Anderson, Jian-Ping Jiang, Wenhao Wu, Neil H. Shubin, Ke-Qin Gao

**Affiliations:** ^1^ School of Earth and Space Sciences, Peking University, 5 Yiheyuan Road, Beijing 100871, China.; ^2^ Department of Comparative Biology and Experimental Medicine, University of Calgary, 3330 Hospital Drive, Calgary, Alberta T2N 1N4, Canada.; ^3^ State Key Laboratory of Palaeobiology and Stratigraphy (Nanjing Institute of Geology and Palaeontology, CAS), 39 East Beijing Road, Nanjing, Jiangsu Province 210008, China.; ^4^ Chengdu Institute of Biology, Chinese Academy of Sciences, Chengdu, Sichuan Province 610041, China.; ^5^ Research Center of Palaeontology and Stratigraphy, College of Earth Sciences, Jilin University, Changchun, Jilin Province 130061, China.; ^6^ Department of Organismal Biology and Anatomy, Biological Sciences, The University of Chicago, Chicago, IL 60637, USA.

## Abstract

Early limb skeletogenesis in salamanders is characterized by preaxial elements, digits I and II forming earlier than their postaxial counterparts (digits III to V), a phenomenon known as preaxial dominance, whereas in amniotes and anurans, these developmental sequences are reversed. This pattern characterizes the late skeletogenesis of digits and zeugopodium of anamniote tetrapods but remains unknown in carpals/tarsals. To correct this gap in knowledge, we investigate the ossification patterns of the carpals/tarsals in six salamander families/clades based on micro–computed tomography scans. We found that preaxial dominance is seen in the distal carpals/tarsals of several salamander clades and diverse early tetrapods, such as temnospondyls and amniotes. This distribution suggests that preaxial dominance is a primitive developmental pattern in tetrapods. Our results demonstrate that the distal carpals/tarsals are developmentally and evolutionarily independent in the autopodium, and preaxial dominance facilitates stabilization of the number of distal carpals/tarsals during fin-to-limb transition and digit reduction in early tetrapods.

## Teaser

Preaxial dominance in the mesopodium is limited to distal carpals/tarsals and facilitates digit reduction in early tetrapods.

## INTRODUCTION

**Fig. 1.**
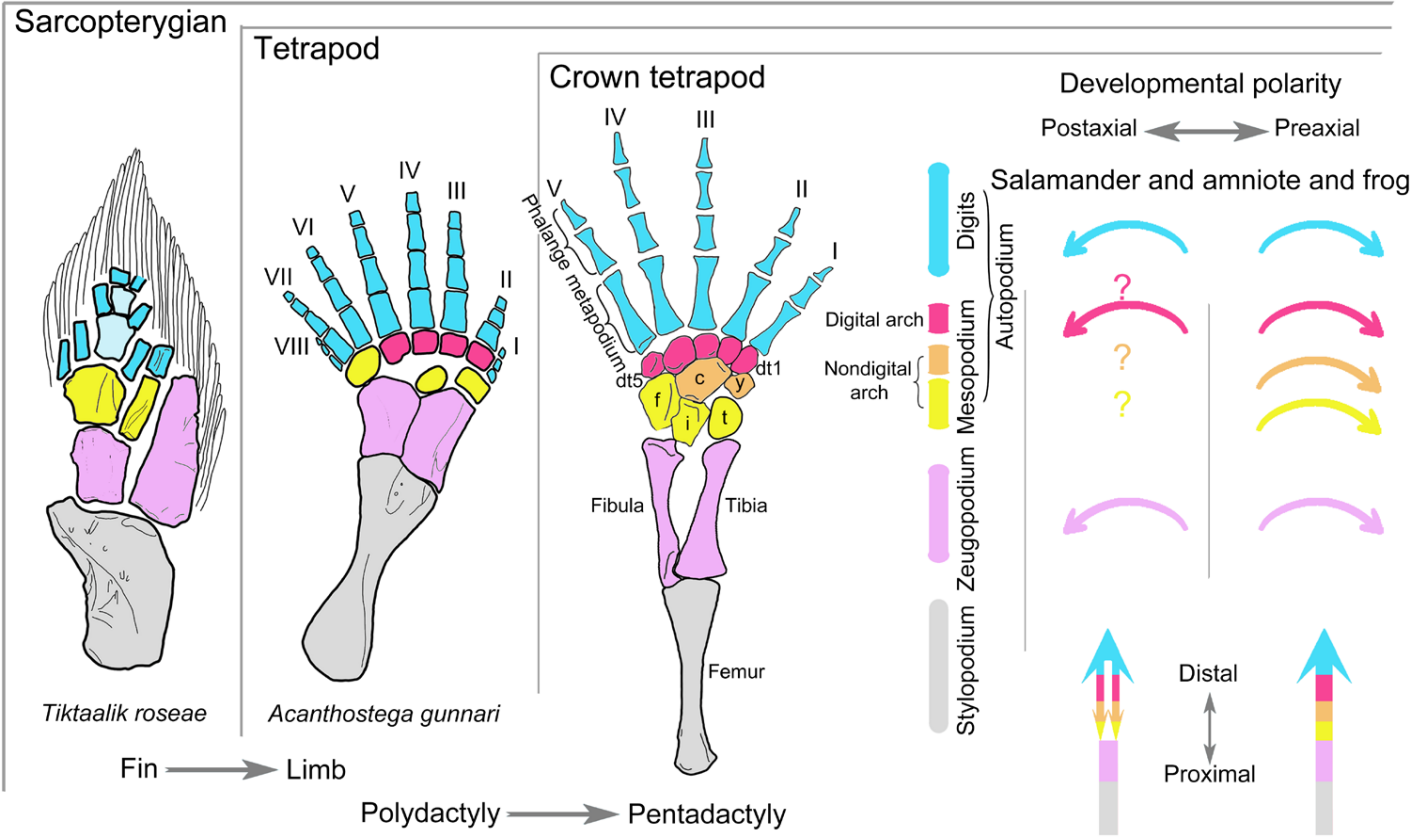
Structural homology between sarcopterygian fins and tetrapod limbs, with comparison of previous understandings on limb developmental sequences during early and late skeletogenesis in salamanders versus that in frogs and amniotes. Note that distal carpals/tarsals 1 and 2 remain separate in the schematic hindlimb of crown tetrapods, but only a single element (basale commune) is present in salamanders and some early tetrapods. Following Gegenbaur’s scheme (*2*, *73*), the mesopodium in the schematic hindlimb of crown tetrapods is divided into three transverse rows in different colors to highlight homologous structures that are present or absent in fish fins: distal (red), central (orange), and proximal (yellow). The main text generally follows Goette’s scheme (see definition in Materials and Methods) (*74*) of dividing the mesopodium into three columns for the ease of developmental analyses. Previous understandings of the developmental patterns of tetrapod limb structures along the anteroposterior and proximodistal axes are denoted by corresponding, color-coded arrows (right figure). *Acanthostega* hindlimb (*7*) and *Tiktaalik* pectoral fin (*9*). c, centrale; dt, distal tarsal; f, fibulare; i, intermedium; t, tibiale; y, element y.

One of the greatest evolutionary transformations in vertebrates is the fin-to-limb transition (*1*–*6*). The limb of tetrapods is composed of the stylopodium (humerus/femur), zeugopodium (radius/tibia on the preaxial side and ulna/fibula on the postaxial side), and the autopodium, which, in turn, consists of the mesopodium (carpals/tarsals) and the digits (metapodium and phalanges; Fig. 1). Homologs for many of these structures have been identified in fish ancestors on the basis of shared phenotypes and articulation patterns [e.g., (*7*–*10*)], molecular regulatory networks, and many gene expression patterns [e.g., (*5*, *6*, *11*–*13*)]. The fish counterparts of distal mesopodials have not yet been found. In most tetrapods, limb structures are patterned sequentially from the stylopodium to the digits along the proximodistal axis during early skeletogenesis (mesenchymal condensation and chondrification). Postaxial elements of the zeugopodium and autopodium in amniotes and anurans form earlier than the corresponding preaxial elements along the anteroposterior axis, commonly known as postaxial dominance (*14*). By contrast, in salamanders, certain elements in the autopodium form in a distal-to-proximal order (e.g., basale commune and metapodials I and II form earlier than intermedium) (*3*, *15*), and preaxial elements form earlier than their postaxial equivalents. The reversed limb developmental pattern along the anteroposterior axis is known as preaxial dominance (Fig. 1) (*14*, *16*, *17*), which has been hypothesized to be the result of derived heterochronic modifications associated with larval adaptations in early limb skeletogenesis of salamanders [e.g., (*15*, *17*–*19*)]. Preaxial dominance is most pronounced in derived salamander clades with free-living pond-type larvae (*14*) but is diminished in taxa with stream-type larvae, direct development (*19*, *20*–*22*), and primitive salamanders (*15*, *18*, *23*–*25*) because these taxa in varying degrees display developmental patterns characteristic of amniotes and anurans (see Discussion).

As the limb of tetrapods proceeds from chondrogenic development to subsequent ossification, the developmental sequences of preaxial and postaxial dominance are typically retained, particularly in anamniote tetrapods (*26*–*30*). Preaxial dominance was identified in the digits and zeugopodium of three species of temnospondyls (*28*, *31*, *32*), leading to a previously unidentified hypothesis that preaxial dominance is not a derived but instead an ancient developmental pattern in temnospondyls, if not Tetrapoda as a whole (*32*–*34*).

Late skeletogenesis of the mesopodium, however, has received scant attention in anamniote tetrapods, because the carpus and tarsus are typically the last limb structures to ossify or remain cartilaginous throughout life, such as that in neotenic salamander families/clades (Amphiumidae, Pancryptobrancha, Proteidae, and Sirenidae) (*17*, *26*, *28*–*30*, *35*–*37*). Specimens in a handful of tetrapod taxa (see Discussion) reveal preaxial dominance in the ossification of distal tarsals of the Early Permian temnospondyl *Gerobatrachus hottoni* (*35*) and postaxial dominance in the mesopodium of amniotes (*28*), anurans (*26*, *37*), and “lepospondyls” (*29*).

Here, we investigate the ossification patterns of and the relative timing of ossification between the carpus and tarsus in six salamander families/clades that span the phylogenetic range of Urodela (crown group salamanders), including Ambystomatidae, Dicamptodontidae, Plethodontidae, Rhyacotritonidae, and Salamandridae in the derived suborder Salamandroidea and Panhynobia (Hynobiidae and stem hynobiids) (*38*) in the primitive suborder Cryptobranchoidea. Among these salamander clades, we increased the sample of fossil and extant taxa in Panhynobia because the carpus and tarsus of panhynobians display some amniote- and anuran-like developmental patterns in early skeletogenesis (see Discussion) (*15*, *18*, *23*). The carpus/tarsus of panhynobians also has numerous plesiomorphic characters (e.g., two centralia) of Urodela and several atavistic features (e.g., element m) of Temnospondyli that are evolutionarily lost in Salamandroidea (*17*, *38*, *39*). We then compared the ossification patterns with the chondrification patterns of the mesopodium established in Ambystomatidae (*14*, *19*, *21*, *25*, *40*, *41*), Dicamptodontidae (*42*), Hynobiidae (*15*, *18*, *23*–*25*, *43*, *44*), Plethodontidae (*19*–*22*, *45*–*47*), and Salamandridae (*14*, *48*–*50*). Analysis of this large sample reveals that amniote- and anuran-like limb developmental sequences are extensive throughout salamanders, and preaxial dominance in the mesopodium is restricted to the distal carpals/tarsals of most salamander clades, temnospondyls, amniotes, and many early tetrapods. Preaxial dominance is neither constrained by multiple factors, including larval adaptations, nor an ancient feature confined within temnospondyls but represents a primitive developmental mode in tetrapods, as had been previously suggested (*28*, *31*–*33*), and its presence in the development of digital arch mesopodials facilitates the elaboration of distal mesopodials during the fin-to-limb transition and digit reduction in early tetrapods.

## RESULTS

**Fig. 2.**
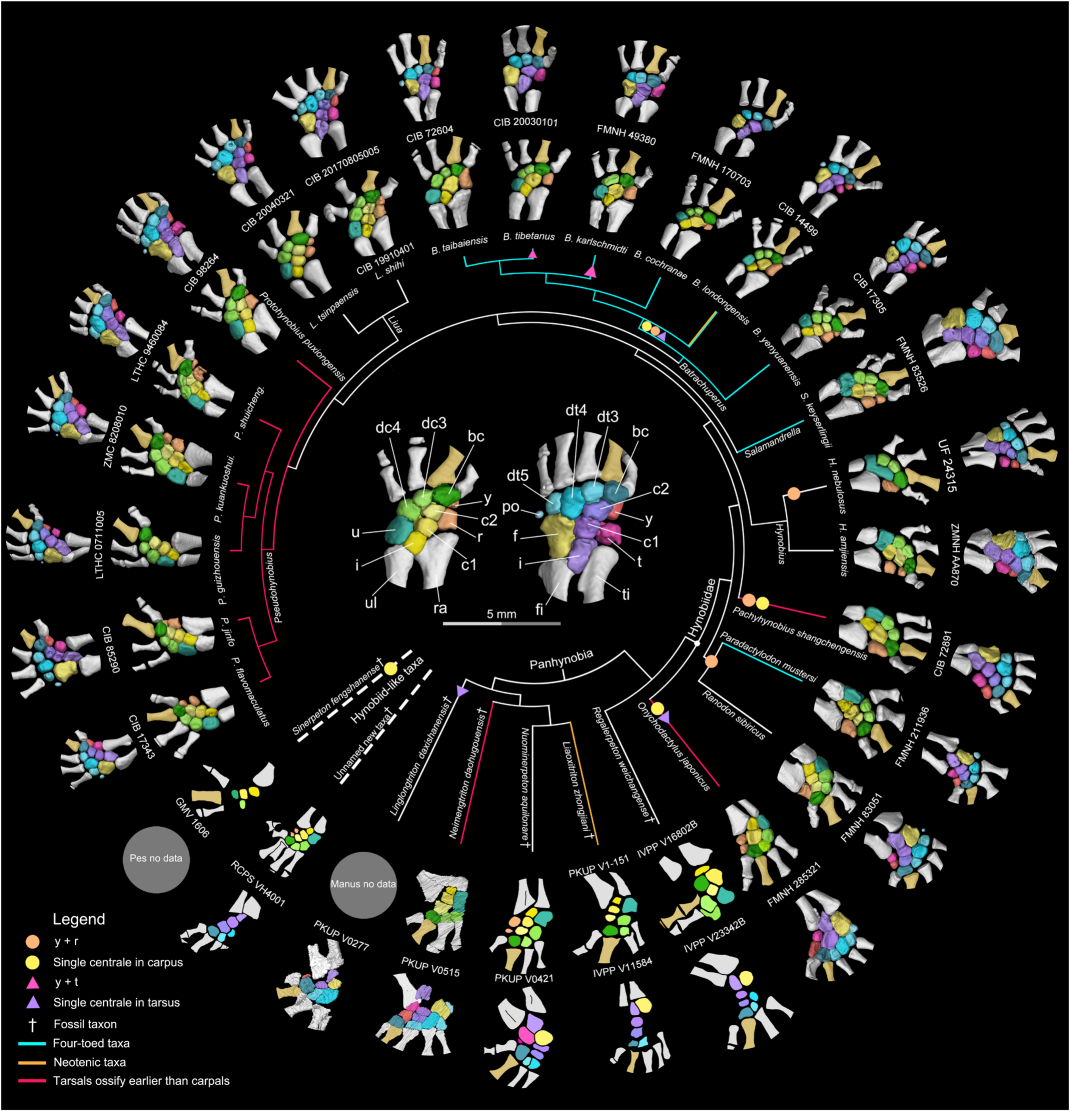
Morphology of the carpus (inner ring) and tarsus (outer ring) in the primitive salamander clade Panhynobia and hynobiid-like fossil taxa showing the prototypical configurations of the mesopodium in salamanders. Supernumerary mesopodials such as extra centrale and postminimus are more commonly present in Panhynobia than previously expected. Note that the preaxial column (element y and radiale/tibiale or their fusions) is located more palmar/plantar than the remaining mesopodials, and element y in particular is usually not fully exposed in dorsal view. The tarsus ossifies earlier than the carpus in *Neimengtriton*, *Onychodactylus*, *Pachyhynobius*, *Protohynobius*, and *Pseudohynobius*, but vice versa in the remaining taxa. Micro–computed tomography (micro-CT)–rendered carpus and tarsus (color-coded, with digit II in gold to differentiate the preaxial side) are based on the largest available specimens in each species. The mesopodium in dorsal view in the center of the cladogram [modified from (*38*, *76*)] is from the holotype [Chengdu Institute of Biology (CIB) 98264] of extant panhynobian *Protohynobius puxiongensis*. Not to scale. See data S1 for details on CT parameters of specimens and data S2 for related biological features of species. bc, basale commune; dc, distal carpal; fi, fibula; po, postminimus; r, radiale; ra, radius; ti, tibia; u, ulnare; ul, ulna; FMNH, Field Museum of Natural History; GMV, Geological Museum of China; IVPP, Institute of Vertebrate Paleontology and Paleoanthropology; LTHC, Liupanshui Normal University; ZMC, Zunyi Medical University; PKUP, Peking University of Paleontological Collection; RCPS, Research Center of Palaeontology and Stratigraphy; UF, Florida Museum of Natural History; ZMNH, Zhejiang Museum of Natural History.

The mesopodium in the Panhynobia has seven to nine ossified carpals and 9 to 11 ossified tarsals in fully grown adult specimens (Fig. 2 and figs. S1 to S4). A detailed summary of the morphology and variation of the ossified salamander mesopodials is beyond the scope of this study, as the exact number of mesopodials in each species depends on three factors, including whether certain neighboring elements are fused or remain separate (e.g., element y and radiale/tibiale, intermedium and ulnare/fibulare, and distal tarsals 3 and 4), the number of digits, and the number of supernumerary elements. Supernumerary mesopodials are more commonly present in Panhynobia than previously appreciated. Two centralia are present in most panhynobian genera except that a single centrale is retained in the carpus of *Onychodactylus*, *Pachyhynobius*, and the Early Cretaceous *Sinerpeton*, as well as in the tarsus of *Onychodactylus* and the Late Jurassic *Linglongtriton*. The postminimus is present in all crown hynobiid genera and the Middle Jurassic stem hynobiid *Neimengtriton*. The rare supernumerary element m was recognized in *Salamandrella* and *Ranodon* (*23*, *43*) and is additionally identified here in *Batrachuperus yenyuanensis*, *Paradactylodon persicus*, and *Protohynobius puxiongensis*. Our results demonstrate that the loss of element m stems from its fusion with the distal tarsal 4 and leads to a proximal extension for the latter into the central row of the tarsus, because such a proximal extension of distal tarsal 4 is absent when element m remains as an independent bone (figs. S3 and S4). An incomplete fusion between these two tarsal elements is manifested by an unfinished notch at the posterior border of distal tarsal 4 (fig. S1).

Elements in the carpus and tarsus have consistent differences in relative size and retain a stable spatial relationship with one another and neighboring zeugopodials and digits despite variation of other morphological features. The basale commune articulates distally with both the metapodials I and II, and the remaining distal carpals/tarsals have a one-to-one articulation with their corresponding metapodials; the postminimus, if present, articulates with the posteriormost distal tarsal and the fibulare. Elements in the preaxial column (element y and radiale/tibiale or their fusion) are located more palmar/plantar than the remaining mesopodials, and in particular, element y is often located anteroventral to the basale commune. The ulnare/fibulare in the postaxial column is the largest carpal/tarsal. In the central column, the centrale in the manus is similar to or slightly larger than its corresponding intermedium, whereas the centrale in the pes is smaller than the corresponding intermedium. The steady size differences and spatial relationship patterns of the mesopodials facilitate identifications of single carpal and tarsal elements in ontogenetic series, fossil specimens, and the derived suborder Salamandroidea, in the latter of which the number of mesopodials is evolutionarily reduced because of loss of supernumerary elements.

### Ossification patterns in the carpus and tarsus in salamanders

**Fig. 3.**
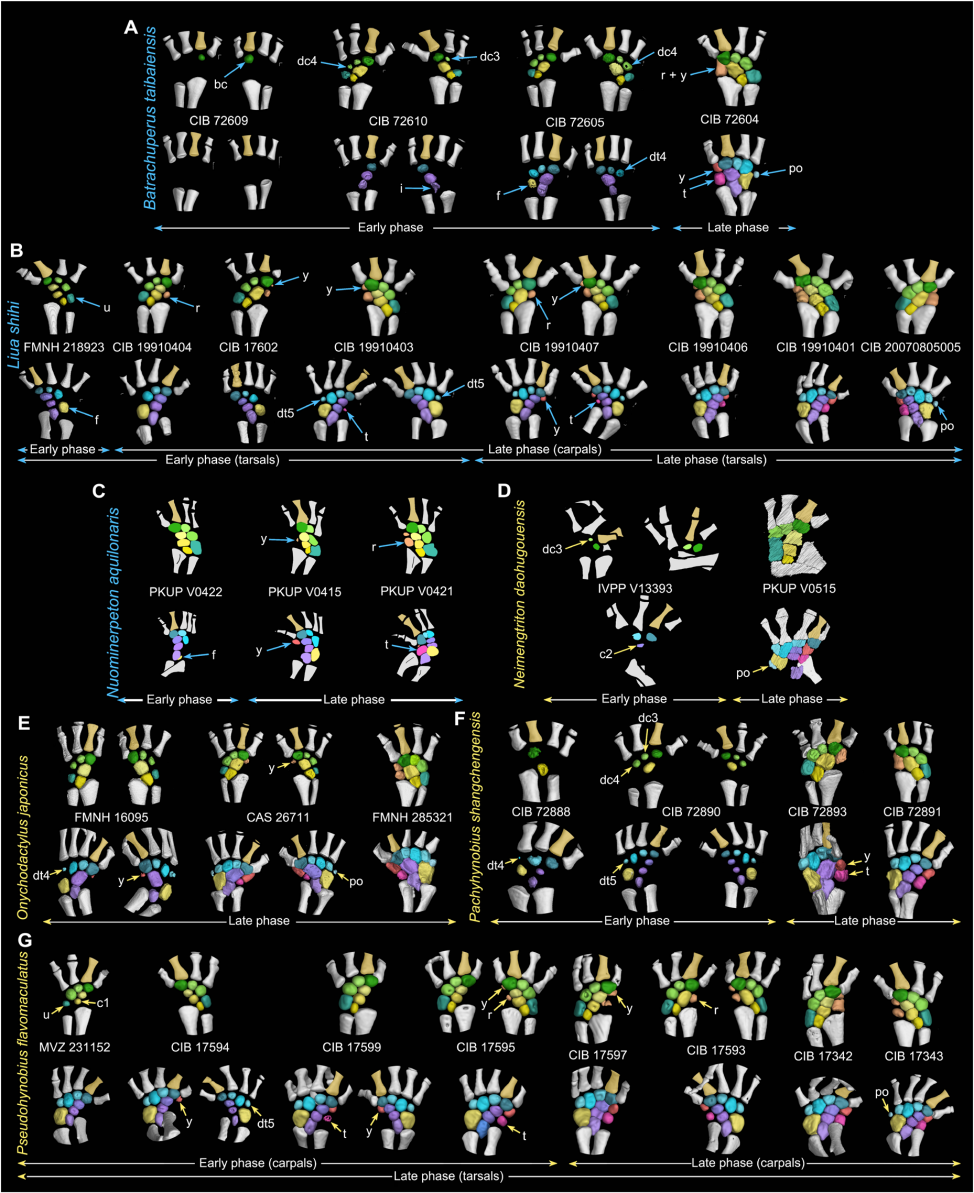
Ossification patterns of the carpus and tarsus in the primitive salamander clade Panhynobia. Specimens are arranged with catalog numbers placed between the carpus (top row) and tarsus (bottom row) in each specimen. Each of the five crown (**A**, **B**, and **E** to **G**) and two stem (**C** and **D**) panhynobians are arranged from left to right to show increased ossification of the mesopodium over ontogeny (not to scale). Most specimens are shown in dorsal view, except three specimens (FMNH 285321, CIB 72893, and CIB 17597) are shown in ventral view to better visualize the mesopodials. Mesopodials are color-coded following Fig. 2, with the preaxial side of the limb denoted by digit II in gold color. The digital arch mesopodials (distal carpals/tarsals and the postminimus) ossify following the preaxial dominance from the basale commune to the postminimus, if any. By contrast, ossifications in nondigital arch mesopodials are characterized by the postaxial dominance, with the preaxial column (element y and radiale/tibiale or their fusions) remaining as the last part to ossify. Along the proximodistal axis, central and preaxial columns generally ossify from distally to proximally with a reversed ossification sequence in the preaxial column present in *Liua shihi* (B) and *Pseudohynobius flavomaculatus* (G). Note that species with names colored in blue have carpus ossified earlier than tarsus and that species with names colored in yellow have carpus ossified later than tarsus.

**Fig. 4.**
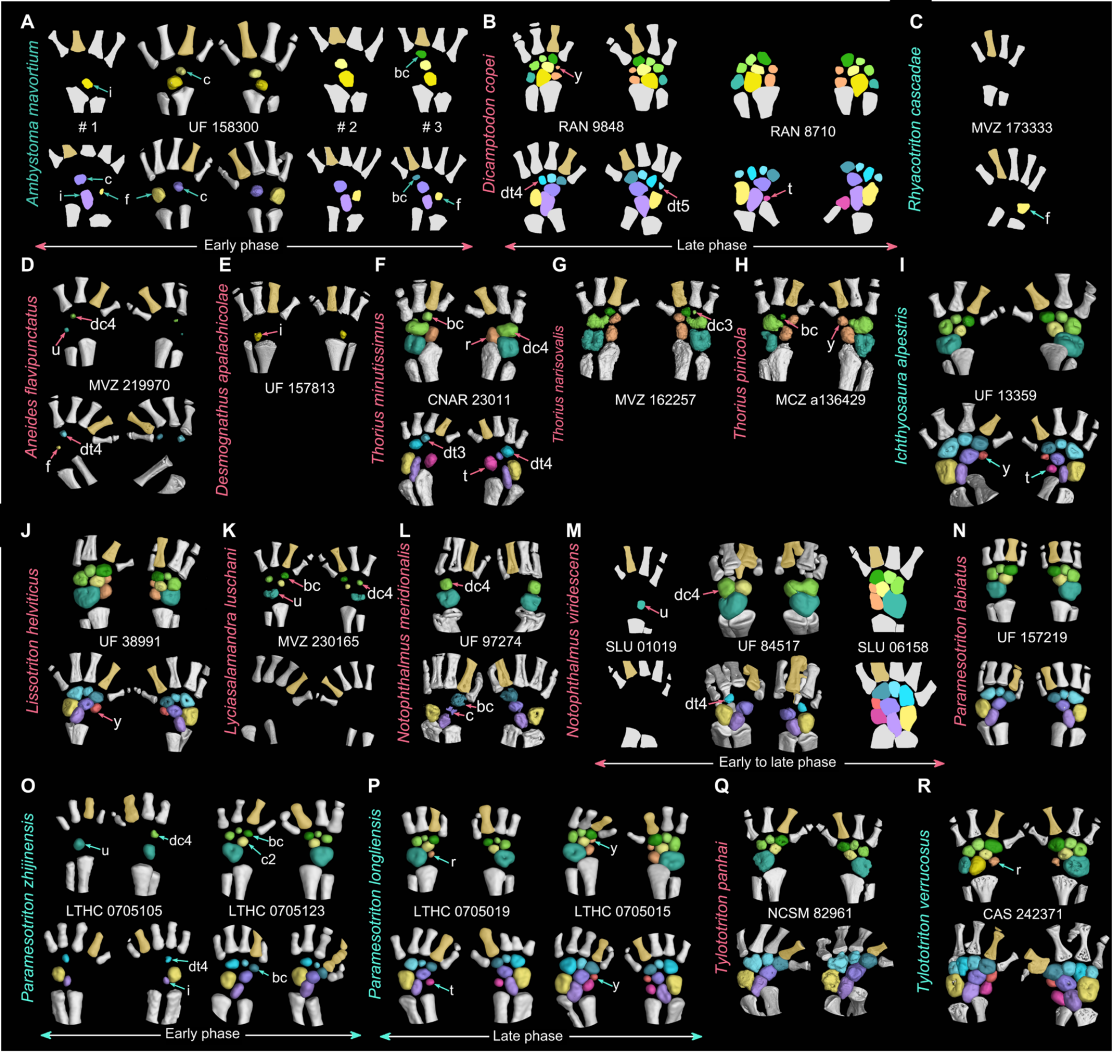
Ossification patterns of the carpus and tarsus in five families of the derived salamander suborder Salamandroidea. In each species of Ambystomatidae (**A**), Dicamptodontidae (**B**), Rhyacotritonidae (**C**), Plethodontidae (**D** to **H**), and Salamandridae (**I** to **R**), specimens [catalog numbers placed between the carpus (top row) and tarsus (bottom row)] are arranged from left to right to show an increased ossification of the mesopodium (not to scale; dorsal view; color-coded following Fig. 2, with digit II colored in gold to differentiate the preaxial side of the limb). Postaxial dominance is present in the ossification of nondigital arch mesopodials with preaxial column as the last part to ossify. In digital arch mesopodials, preaxial dominance is present in Ambystomatidae (A), Dicamptodontidae (B), and *Notophthalmus meridionalis* (tarsus only) (L) but is reversed in Plethodontidae (D and F to H) and Salamandridae (K to M and O), where distal carpal/tarsal 4 ossifies earlier than basale commune. Along the proximodistal axis, mesopodials ossify from proximally to distally in most taxa, with a reserved ossification sequence found in the preaxial column of two salamandrids (I and J). Note that species with names colored in cyan have carpus ossified later than tarsus and that species with names colored in red have carpus ossified earlier than tarsus. CAS, California Academy of Sciences; CNAR, National Autonomous University of Mexico; MCZ, Museum of Comparative Zoology; MVZ, Museum of Vertebrate Zoology; NCSM, North Carolina Museum of Natural Sciences; RAN, Ronald Nussbaum Collections at Museum of Zoology; SLU, Southeastern Louisiana University Vertebrate Museum.

The preaxial column and the postminimus, if any, are always the last mesopodials to ossify in all salamanders investigated (Figs. 3 and 4 and figs. S1 to S5). The delay in ossification between the preaxial column and the remaining mesopodials except the postminimus is extraordinary, because the preaxial column can remain unossified up to six to nine years, as documented in *Salamandrella keyserlingii* (*18*), and the time it takes for full ossification of the preaxial column in our sample covers a growth of up to 40 mm in the snout-pelvic length (data S1). We therefore divided the ossification processes of the mesopodium into an early and late phase, with the onset of ossification of the preaxial column and the postminimus marking the commencement of the late phase, whereas that of all remaining mesopodials occur in the early phase. On the basis of available specimens and comparisons of the size and ossification extent among mesopodials, we found that the ossification sequences are variously polarized within the digital arch and each of the postaxial, central, and preaxial columns.

In the digital arch of the mesopodium, ossification proceeds anteroposteriorly from the basale commune toward the posteriormost carpal/tarsal in the early phase and concludes in the late phase when the postminimus is ossified. Such a preaxial polarized ossification pattern is observed in the digital arch of eight panhynobian genera (*Batrachuperus*, *Liaoxitriton*, *Liua*, *Neimengtriton*, *Nuominerpeton*, *Onychodactylus*, *Pachyhynobius*, and *Pseudohynobius*), two hynobiid-like taxa (*Sinerpeton* and an unnamed new taxon; Figs. 2 and 3), and two families (Ambystomatidae and Dicamptodontidae) of Salamandroidea. However, ossification in the digital arch mesopodials is reversed in many taxa of Plethodontidae (*Aneides*, *Thorius*, and probably *Karsenia*) and Salamandridae (*Notophthalmus*, *Paramesotriton*, *Pleurodeles*, *Taricha*, and probably *Lyciasalamandra*; Fig. 4 and fig. S5), in which the distal carpal/tarsal 4 ossifies first and is followed sequentially by the basale commune and distal carpal/tarsal 3 (data S2). In the postaxial column, the ulnare/fibulare ossifies later than the distal carpals/tarsals 3 and/or 4, and the fibulare ossifies earlier than both the distal tarsal 5 and the postminimus in panhynobians (*Batrachuperus*, *Liua*, *Nuominerpeton*, *Onychodactylus*, *Pachyhynobius*, and *Pseudohynobius*). The fibulare in *Dicamptodon* ossifies earlier than distal tarsal 5 as in panhynobians, whereas the ulnare/fibulare ossifies simultaneously with distal carpal/tarsal 4 in Plethodontidae (*Aneides* and *Karsenia*), earlier than distal carpals/tarsals 3 and 4 in Ambystomatidae and Plethodontidae (*Thorius*), or represents the first carpal/tarsal to ossify in Rhyacotritonidae and Salamandridae (*Notophthalmus*, *Paramesotriton*, and *Pleurodeles*). Ossifications in the central column proceed distoproximally from the centrale toward the intermedium in panhynobians (*Batrachuperus*, *Liua*, *Neimengtriton*, *Pachyhynobius*, *Protohynobius*, and *Pseudohynobius*), with the centrale ossifying later than, or perhaps simultaneously with, the basale commune (*Batrachuperus*, *Neimengtriton*, and *Pachyhynobius*), whereas the intermedium, the centrale, and the basale commune ossify successively in Ambystomatidae, Plethodontidae (*Desmognathus*, *Karsenia*, and *Thorius*), and Salamandridae (*Notophthalmus*, *Paramesotriton*, and *Pleurodeles*).

In the late phase, opposite ossification sequences within the preaxial column are also observed in taxa where element y and radiale/tibiale are separate from each other. Most panhynobians have element y ossify earlier than the radiale/tibiale, but a reversed polarity is found as a standard pattern in *Liua shihi* or as an intraspecific variation in *Pseudohynobius flavomaculatus* and *B. yenyuanensis*. By contrast, preaxial column ossifies proximodistally in most taxa of Salamandroidea, and that in only a few taxa have the element y ossify earlier than radiale/tibiale in Plethodontidae (*Bolitoglossa*) and Salamandridae (*Ichthyosaura*, *Lissotriton*, and *Pleurodeles*). The postminimus is the final tarsal to ossify.

### Relative ossification sequences between the carpus and tarsus

The carpals ossify earlier than the corresponding tarsals in most panhynobian genera, Dicamptodontidae, Plethodontidae (*Bolitoglossa*), and Salamandridae (*Lissotriton*, *Lyciasalamandra*, *Notophthalmus*, and *Pleurodeles*), whereas the tarsals ossify earlier than the carpals in five panhynobian genera (*Neimengtriton*, *Onychodactylus*, *Pachyhynobius*, *Protohynobius*, and *Pseudohynobius*), Ambystomatidae, Rhyacotritonidae, Plethodontidae (*Aneides*), and Salamandridae (*Ichthyosaura*, *Notophthalmus*, *Ommatotriton*, *Paramesotriton*, and *Tylototriton*).

## DISCUSSION

The divergent patterns in chondrification during early limb skeleton formation in salamanders (*2*) historically led to the idea of a diphyletic origin of tetrapods, where salamanders were hypothesized to be more closely related to dipnoans or porolepiform fishes than any other tetrapods (*3*, *51*, *52*). Another hypothesis was that salamanders had experienced a change in digit identities during early evolution, where the “original” digits I and II were lost and replaced by elements homologous to digits III and IV of amniotes and anurans, with the “current” digits III to V emerging as de novo elements (*53*).

Historically, our understanding of salamander limb development is largely based on species that underwent metamorphosis with pond-dwelling larvae, including *Ambystoma mexicanum* (*3*, *14*, *17*, *19*, *21*, *25*, *40*, *48*), *Taricha granulosa* (*50*), “*Triton*” (synonym to *Triturus* and/or *Lissotriton* depending on species) (*3*, *48*), and *Triturus marmoratus* and *Triturus boscai* (*49*). As best documented in *T. marmoratus*, the basale commune and metapodials I and II form first and lack continuous condensations connecting them to more proximal mesopodials; the basale commune segments proximally to form the centrale and the intermedium, and posteriorly to give rise to the distal carpal/tarsal 3; the radiale/tibiale forms earlier than the intermedium and the ulnare/fibulare; and then the radiale/tibiale segments distally to form element y, the ulnare/fibulare segments distally to form distal carpal/tarsal 4, and the distal tarsal 4 segments to form distal tarsal 5. Relatively early formation of the basale commune and metapodials I and II in development is observed in every taxon above, whereas the timing of the formation of nondigital arch mesopodials remains unclear in “*Triton*” and *Taricha*. The nondigital arch mesopodials in *T. boscai* form from the preaxial to the postaxial side, whereas the central column forms first in *A. mexicanum* (termed “central polarity” in some studies) and is followed successively by the preaxial and postaxial columns. Along the proximodistal axis, formation of the central column proceeds from the intermedium to the basale commune in *Ambystoma* and *Taricha* but is reversed from the basale commune to the intermedium in *Triturus* (*17*, *50*).

More recently, studies on the early skeletogenesis of the mesopodium have been extended to nine other species of three salamander clades, including Dicamptodontidae (*Dicamptodon tenebrosus*), Plethodontidae (*Bolitoglossa subpalmata*, *Desmognathus aeneus*, *Desmognathus quadramaculatus*, and *Plethodon cinereus*), and Panhynobia (*Hynobius*, *Onychodactylus*, *Ranodon*, and *Salamandrella*). All of these species form basale commune and metapodials I and II relatively early in development as in *Triturus* but variously display developmental patterns characteristic of amniotes and anurans. For example, the intermedium forms first and is followed sequentially by the ulnare/fibulare and the radiale/tibiale in the stream-type larvae of *D. tenebrosus* (*42*), in the primitive hynobiid *Salamandrella* (*15*, *18*, *43*, *54*), and the plethodontid *D. quadramaculatus* (*20*, *47*). Along the proximodistal axis of the limb, the proximal mesopodials (e.g., intermedium) form either earlier than (*Hynobius*, *Andrias*, *Cryptobranchus*, *Dicamptodon*, *Salamandrella*, *D. aeneus*, and *T. granulosa*) (*3*, *17*, *20*, *21*, *28*, *50*) or simultaneously with (*Ranodon*) (*23*, *24*) the distal mesopodials (e.g., basale commune). An amniote-like continuous condensation connecting the postaxial column of the mesopodium with the digital arch was found in at least two plethodontids, *Bolitoglossa* and *Plethodon* (*19*, *22*, *25*, *46*).

**Fig. 5.**
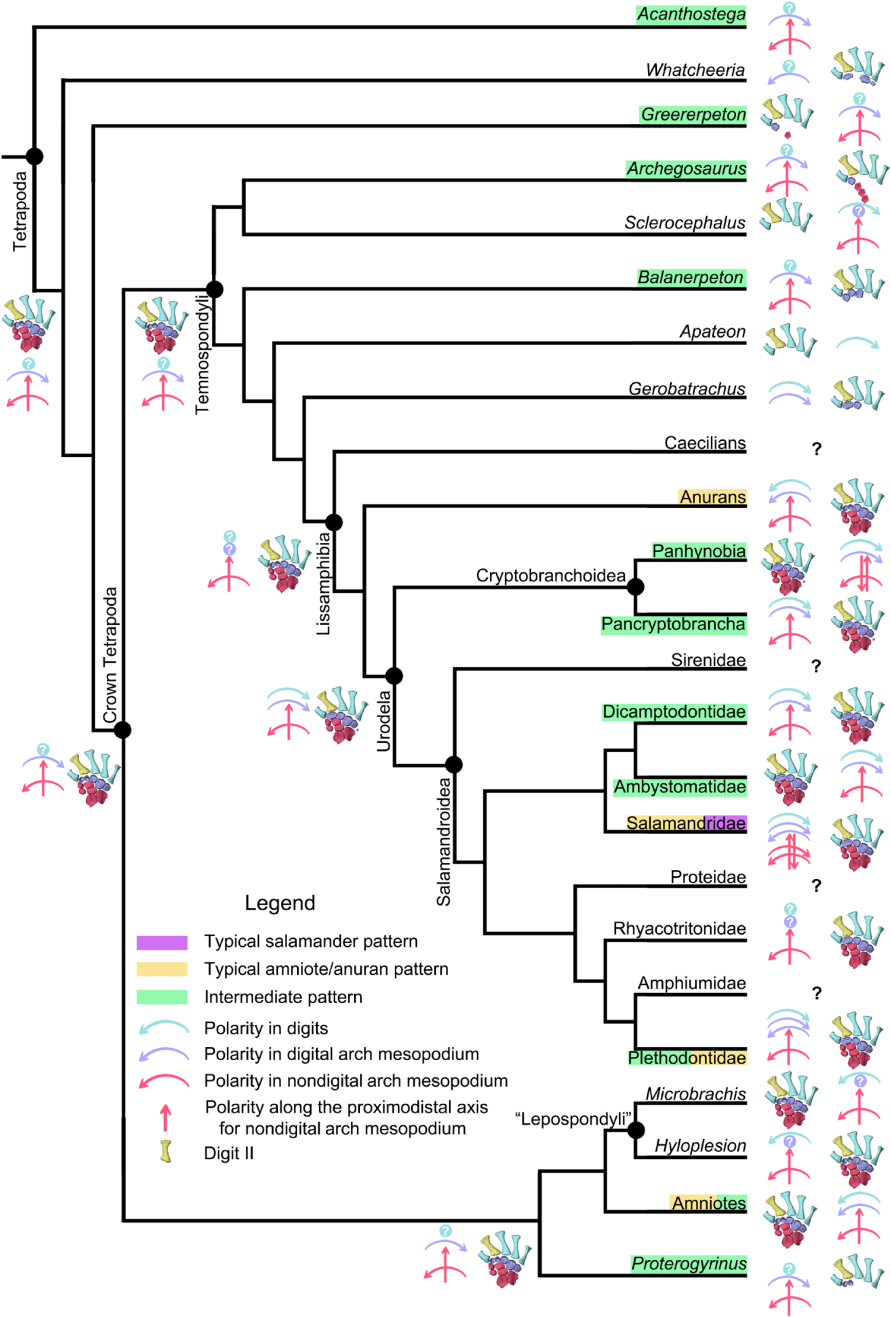
Evolution of the developmental patterns (early and late skeletogenesis) of the autopodium in tetrapods. Taxa with names framed in purple rectangles have typical salamander preaxial dominance across the mesopodium, those in orange have typical amniote/anuran postaxial dominance across the mesopodium, and those in green have a mixed “intermedium” mode with preaxial dominance in the digital arch and postaxial dominance in nondigital arch mesopodium. Taxa with names not framed in colorful rectangles have unknown developmental patterns (labeled by colorful solid circles with white “?” in the middle) in the mesopodium, or the mesopodium is absent or remains permanently cartilaginous (collectively by “?” in black). The preaxial side of the autopodium is denoted by metapodial II in gold, and color-coded arrows are used to manifest the developmental polarities along the anteroposterior axis in digits (light blue), digital arch (purple), and nondigital arch (red horizontal) mesopodials and that along the proximodistal axis (red vertical) in preaxial and central columns.

Our results reveal substantial amniote- and anuran-like features during late stages of skeletogenesis of the salamander mesopodium (Fig. 5). Postaxial dominance is consistently present in nondigital arch mesopodials of all investigated salamander clades with the preaxial column (element y and radiale/tibiale) remaining as the last part to ossify. In the digital arch mesopodials, postaxial dominance is also present in Plethodontidae and Salamandridae, where distal carpal/tarsal 4 ossifies earlier than the basale commune. The carpals/tarsals in the preaxial and central columns ossify from proximally to distally in most taxa of Salamandroidea and several derived panhynobians (*Batrachuperus*, *Liua*, and *Pseudohynobius*). These results bridge the gap between salamanders and other modern tetrapods, amniotes and anurans, and reinforce the existence of a general bauplan in tetrapod limb development (*16*, *55*).

**Fig. 6.**
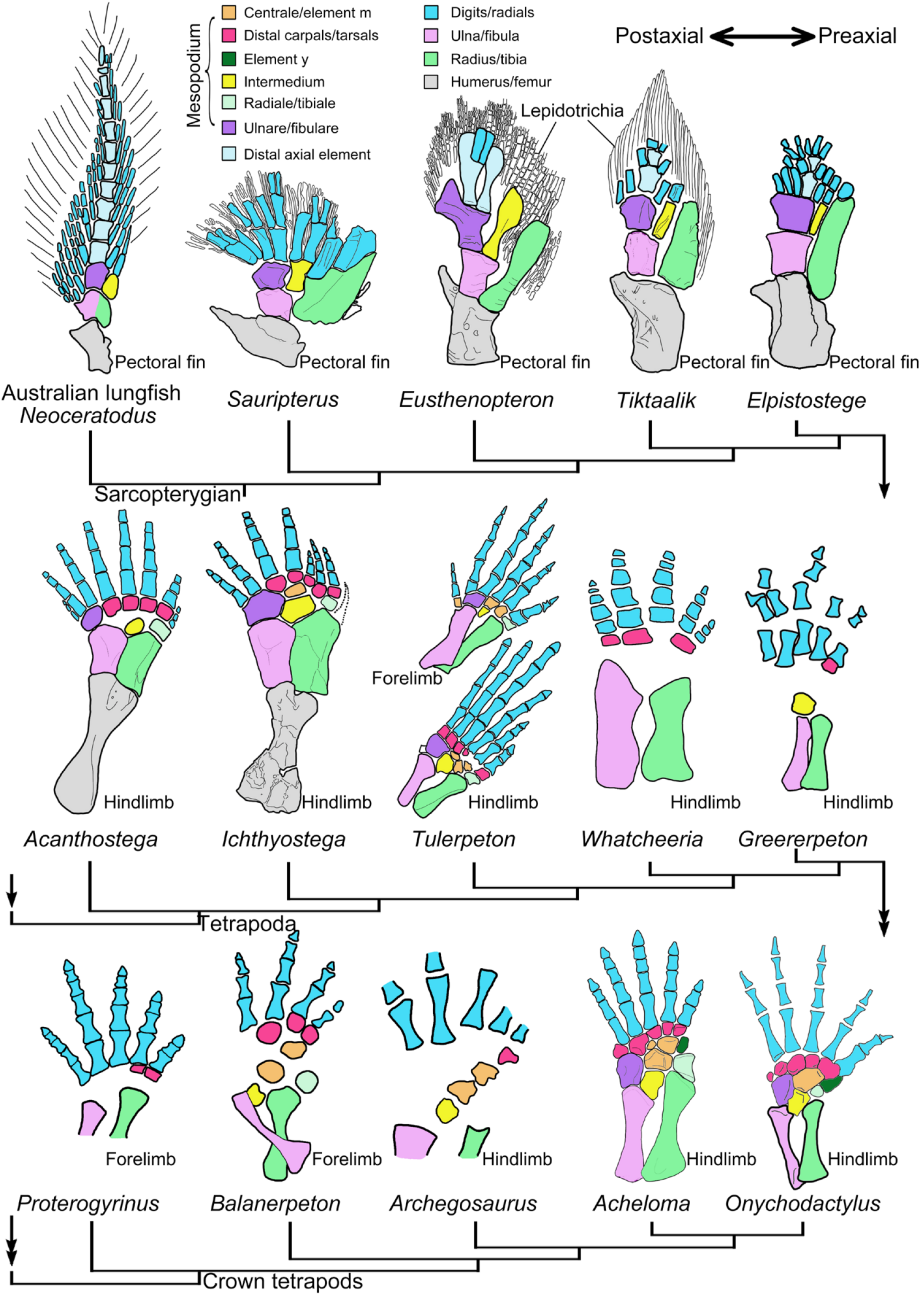
Origin, evolution, and development of the distal carpals/tarsals during the fin-to-limb transition. The ulnare/fibulare articulates directly with most of the proximal radials in tetrapodomorph sarcopterygians *Eusthenopteron*, *Tiktaalik*, and *Elpistostege*, with some postaxial digits in stem tetrapods *Acanthostega* and *Ichthyostega* and with some postaxial distal carpals/tarsals in crown tetrapods. Digital arch mesopodials have an independent evolutionary history and developmental trajectory in the autopodium, as evidenced by the opposite sequences in the loss of distal carpals/tarsals and corresponding digits in tetrapod groups. Distal carpals/tarsals at the postaxial side are lost earlier than digits in early tetrapods (e.g., *Acanthostega*) but later than digits in modern tetrapods (e.g., *Onychodactylus*) when the postminimus is considered as a vestigial digit (see main text). Preaxial dominance facilitates establishing a one-to-one relationship between distal mesopodials and digits in modern tetrapods by promoting the loss of the postaxial distal carpals/tarsals and their associated supernumerary digits in early tetrapods. Interpretations of the radials and distal axial elements along the metapterygial axis follow (*10*, *11*). *Neoceratodus* from (*6*, *11*); *Sauripterus*, *Eusthenopteron*, and *Tiktaalik* from (*9*); *Elpistostege* from (*10*); *Acanthostega* and *Ichthyostega* from (*7*, *11*); *Tulerpeton* from (*70*); *Whatcheeria* from (*68*); *Greererpeton* from (*64*); *Proterogyrinus* from (*65*); *Balanerpeton* from (*63*); *Archegosaurus* from (*61*); *Acheloma* from (*36*); and *Onychodactylus* from CAS 26711.

Comparison of chondrification and ossification patterns of the mesopodium across salamander clades also reveals that developmental sequences are more evolutionarily stable along the anteroposterior axis than along the proximodistal axis. Reversals along the proximodistal axis are widely present in both Panhynobia and Salamandroidea, whereas reversals along the anteroposterior axis are limited only to a few taxa in Salamandroidea. Reversals are common in developmental sequences along the proximodistal axis within the autopodium not only in salamanders but also in many other tetrapod groups. For example, the mesopodium ossifies later than the digits in many extant and fossil amphibians and reptiles [e.g., (*28*, *36*, *56*)]. The proximal-to-distal sequence of development is accepted by most studies as the plesiomorphic mode in tetrapods because it is present in the development of fins in sarcopterygian fish (*11*) and in the limb in amniotes, anurans, and early tetrapods (Figs. 5 and 6) (*7*, *8*, *14*, *18*, *29*).

Classically, the preaxial dominance was argued to be related to the evolution of extracapsular development of limb buds and concomitant functional demands for locomotion while the limb bud is growing [e.g., (*46*)]. In pond-type larvae, these functional demands were claimed to be fulfilled either by an earlier formation and elongation of the digit II in *Triturus* or an earlier formation of morphological specializations in living panhynobians, such as an epidermal fin (a fin-like interdigital membrane) between digits I and II in *S. keyserlingii*. In other words, the functional demand on the digits of *Triturus* is now borne by the epidermal fin in living panhynobians, and as a result, postaxial and proximal mesopodials in the latter taxa, such as ulnare/fibulare, form early as in other tetrapods [e.g., (*15*, *18*, *23*, *42*, *54*)]. In other types of salamander larvae, these selective pressures from locomotion were argued to diminish or disappear in stream-living larvae of Dicamptodontidae and the direct-developing Plethodontidae, because similar to amniotes and anurans, their limb buds are not used for locomotion while developing. Instead, limb development in these taxa is achieved intracapsularly, facilitated by their large egg size and extended embryonic period [e.g., (*20*, *42*, *46*)]. Hence, the preaxial dominance was widely interpreted as a derived developmental mode that is modified from a general bauplan in tetrapod limb development.

Similarly, larval types and life history strategies have been proposed to be related to the developmental timing of fore- and hindlimbs: The forelimb bud develops earlier than the hindlimb bud in species with pond-type larvae that undergo metamorphosis [e.g., *Ambystoma* in (*40*), *Triturus* in (*49*), and *Salamandrella* in (*18*)], while such a difference is less pronounced in metamorphosed species with stream-type larvae [e.g., *Dicamptodon* in (*42*), *Onychodactylus* in (*57*), and *Hynobius* in (*58*)]. Differences in the timing of the development of fore- and hindlimbs are absent in direct developing Plethodontidae, in which the hindlimb bud appears either simultaneously with or earlier than the forelimb bud [e.g., (*20*, *46*, *47*)]. Our results, however, demonstrate that the traditional explanations on the preaxial dominance and relative developmental timing between manus and pes in early skeletogenesis are not applicable to late skeletogenesis of the mesopodium. Rather, species characterized by preaxial dominance in the mesopodium or those taxa with either carpus or tarsus leading in ossification sequences not only occupy the full phylogenetic spectrum of Urodela but also are diversified in larval types, life history strategies, and ecological preferences (data S2). Our observation indicates that the late skeletogenesis of mesopodium is free from these potential constraints.

Possible constraints on the preaxial dominance in distal mesopodials are biomechanical demands for locomotion in adult life—mesopodials begin to ossify after sexual maturity is reached in salamanders (*28*). Biomechanical stresses from muscle contractions may likely influence ossification sequences in salamander limb structures as that in therian mammals (*59*). In therians, marsupials uniquely have their scapula ossify earlier than femur, their carpals ossify simultaneously with tarsals when compared to placentals, and the accelerated ossification of forelimb bones was argued as most likely caused by accelerated development of shoulder muscles and the functional demands for the young to hold the mother’s fur while within the pouch (*59*). However, in salamanders, the way in which biomechanical distributions affect the relative ossification sequences of mesopodials remains unclear because of limited studies on the arrangements, sequences of activities in musculature, and stress distribution in the mesopodium.

We agree with Schmalhausen (*54*) that the delay in the development of the preaxial column in salamanders is caused by its independence in the mesopodium. By investigating early limb skeletogenesis in living panhynobians, he and other researchers noticed that the element y and radiale/tibiale are separated by the central artery from the remaining mesopodials (*43*, *60*), and fusions of neighboring elements between the preaxial and central columns rarely happen in salamanders [e.g., (*18*)]. Our results found additional support for the independence of preaxial column in the mesopodium—element y and radiale/tibiale (i) are located toward the anteroventral border of the central column and do not share the same palmar/plantar plane with the remaining mesopodials, (ii) are always the last mesopodials to ossify with a prolonged delay, and (iii) lack fusions with elements in the central column.

A recent molecular study (*34*) demonstrates that the preaxial dominance in tetrapod limb development is controlled by the activity level of *Gli3* repressor and the expression of 5′ *Hoxd* genes, and a switch from postaxial to preaxial dominance in mice or that from preaxial to postaxial dominance in *Ambystoma* can be managed by enhancing *Gli3* repressor activity and *Gli3* knockdown, respectively. Given the variability of development in salamanders, it will be interesting to see the ways molecular features covary with morphological patterns of development.

The evidence from diverse salamander limbs does not support interpretations that preaxial dominance either represents a derived developmental pattern in tetrapods [e.g., (*18*, *19*)] or has an evolutionary history confined within temnospondyls (*32*, *33*), although in the latter studies (*32*, *33*), preaxial dominance was suggested as a possible plesiomorphic state in tetrapod limb development. Instead, our data support the notion that preaxial dominance is a primitive developmental pattern for the digital arch mesopodials in tetrapods (Fig. 6). Before this current study, preaxial dominance was found in the ossification of digits of three temnospondyls *Sclerocephalus haeuseri*, *Micromelerpeton credneri*, and *Apateon* sp. and in the ossification of digital arch mesopodials of a fourth temnospondyl, *G. hottoni*, in which the holotype specimen has a basale commune and distal tarsal 3 with the former being larger than the latter (*35*). Here, we found that distal carpals/tarsals on the preaxial side are always the only ossified mesopodials in the digital arch in at least three more temnospondyls, *Archegosaurus decheni* (distal carpal/tarsal 1) (*61*), *Amphibamus lyelli* (distal tarsals 1 to 3) (*62*), and *Balanerpeton woodi* (distal carpals 1 to 3) (*63*), one nectridean “lepospondyl” *Sauropleura scalaris* (distal tarsals 1 to 3) (*62*), one basal amniote *Diadectes* sp. (distal tarsals 1 and 2) (*62*), and two early tetrapods from the Carboniferous, *Greererpeton burkemorani* (dt 1) (*64*) and *Proterogyrinus scheelei* (distal carpals 1 and 2) (*65*). The phylogenetic positions of these two Carboniferous tetrapods are unstable; *Greererpeton* was recovered as closely related to either the basal crown or stem tetrapods, and *Proterogyrinus* is widely accepted as affiliated to Embolomeri, which, in turn, was argued as an order of crown or stem tetrapods or Reptiliomorpha [e.g., (*66*, *67*)]. An even more primitive stem tetrapod, *Whatcheeria deltae*, was recently shown to have ossified distal tarsals 1, 4, and 5, with distal tarsal 4 being the largest in the digital arch (*68*). Such a size difference in the digital arch of *Whatcheeria* likely indicates the existence of postaxial dominance, because the distal carpal/tarsal 4 is typically the first to ossify in amniotes and anurans and remains larger than other distal carpals/tarsals during development (*28*, *37*). The preaxial dominance is present in the fin development of the Australian lungfish *Neoceratodus*, in which the preaxial fin radials form earlier than postaxial fin radials following a corresponding anterior-to-posterior shift in expression patterns of *Hoxd13* (*11*). On the other hand, early tetrapods [*Acanthostega* in (*7*), *Ossinodus* in (*69*), *Panderichthys* in (*8*), *Archegosaurus*, *Greererpeton*, and possibly *Whatcheeria*; Fig. 6] and the Australian lungfish (*11*) share with modern tetrapods in having the postaxial dominance characterized the ossification of nondigital arch mesopodials, in which the intermedium and ulnare/fibulare ossify earlier than the radiale/tibiale.

More ontogenetic series and more robust phylogenetic relationships among early tetrapods are needed to fully evaluate the distribution, polarity, and times of origin of the preaxial dominance in the digital arch mesopodials across tetrapod groups. Nevertheless, our results demonstrate that the digital arch mesopodials have a different, or at least a less stable, developmental sequence than the nondigital arch mesopodials in the early evolution of tetrapods. The developmental disparity between digital arch and nondigital arch mesopodials highlights that the mesopodium should not be treated as a single module as is commonly done in molecular studies, in which the autopodium as a whole was argued as homologous to the postaxial fin radials of the Australian lungfish (*6*) or distal fin radials of zebrafish (*12*, *13*). Instead, the digital arch mesopodials must have an independent evolutionary history within the autopodium that may be linked to their enigmatic origin during the fin-to-limb transition.

As the only limb structures with no morphological homologs in fish relatives [e.g., (*8*–*10*)], distal carpals/tarsals were hypothesized to be evolutionary novelties of tetrapods (*25*). Potential carpals were recently claimed to be found in the tetrapodomorph sarcopterygian *Elpistostege* and were argued as to correspond to the distal and central rows of the carpus in modern tetrapods (*10*). However, it is important to note that most of these “potential carpals” in *Elpistostege* articulate directly with the ulnare, and a similar configuration is found in more primitive *Eusthenopteron* and *Tiktaalik*, where the ulnare/fibulare articulates directly with most of the fin radials (Fig. 6). However, these potential carpals that are in direct articulations with the ulnare/fibulare in sarcopterygian fish are evolutionarily lost in the earliest known stem tetrapods (e.g., *Acanthostega* and *Ichthyostega*), because, in the stem tetrapods, the fibulare has a direct articulation with its corresponding digits and have no distal tarsals in between.

In stem tetrapods, the number of distal carpals/tarsals fluctuates across taxa, and here, we argue that a one-to-one correlation between distal carpals/tarsals in modern tetrapods is likely achieved by the preaxial dominance that characterizes the development of digital arch mesopodials. On the basis of specimens with the highest amount of ossification in the mesopodium, the number of distal carpals/tarsals [4-5-0(6)-5] in the three most primitive stem tetrapods are in conflict with digit reduction (8-7-6-5). *Acanthostega* (eight digits) has four distal tarsals and lacks the posteriormost three, and possibly the anteriormost distal tarsals, assuming that its limb skeleton was correctly restored (*7*). *Ichthyostega* (seven digits) has five distal tarsals and lacks the posteriormost two distal tarsals (*7*). In the more crownward *Tulerpeton* (six digits), the story is more complicated. All distal carpals are not present in the only specimen that shows this region, but there are five ossified distal tarsals articulating with their corresponding toes, except that distal tarsal 2 remains cartilaginous or is not preserved (*70*). As exemplified by the many last-to-form and first-to-lose cases in cranial bones, loss of distal carpals/tarsals on the postaxial side of the digital arch in these early stem tetrapods is likely facilitated by their late formation during development. The loss of distal carpals/tarsals, in turn, may induce the loss of corresponding supernumerary digits in early tetrapods. In modern salamanders, however, the loss of distal mesopodials instead lags behind the loss of corresponding digits, because the distal carpal/tarsal 4 is normally present in a variant of the three-toed *Amphiuma tridactylum* (*41*), a three-toed variant of *B. yenyuanensis*, or when digit IV was experimentally inhibited to form in *A. mexicanum* (*71*), and the postminimus in the digital arch represents a vestige of an additional digit in primitive salamanders such as panhynobians (*72*). The conflicting developing timing of distal carpals/tarsals and corresponding digits between salamanders and early tetrapods, as apparently mediated by heterochrony, strengthen our arguments that the distal carpals/tarsals have an independent evolutionary history and developmental trajectory within the autopodium, and the preaxial dominance in the digital arch mesopodials facilitates stabilizing the number of distal mesopodials during the fin-to-limb transition and digit reduction from polydactyly to pentadactyly in early tetrapods.

## MATERIALS AND METHODS

### Experimental design and specimens

All six families/clades of modern salamanders with ossified carpus and tarsus were examined here on the basis of a total of 200 living and 14 fossil specimens that represent 60 species in 37 genera: Ambystomatidae (6 specimens for 3 species in *Ambystoma*), Dicamptodontidae (5 specimens for 2 species in *Dicamptodon*), Panhynobia (169 specimens), Plethodontidae (8 specimens for 7 species in 5 genera), Rhyacotritonidae (2 specimens for 2 species in *Rhyacotriton*), and Salamandridae (24 specimens for 15 species in 11 genera). In particular, our specimen sampling covers all genera of the primitive salamander clade Panhynobia, including 155 specimens for 23 living species in 10 genera of Hynobiidae (crown Panhynobia) and 11 fossil specimens for 5 species in 5 stem hynobiid genera (*Liaoxitriton*, *Linglongtriton*, *Neimengtriton*, *Nuominerpeton*, and *Regalerpeton*) that were found from the Middle Jurassic to Lower Cretaceous of northern China. We also included a specimen each for two hynobiid-like fossil taxa (*Sinerpeton* and *Laccotriton*) and a specimen for an unnamed new taxon from the Lower Cretaceous of Hebei Province, China (data S1 and S2). A total of 36 specimens were accessed through two online platforms: MorphoSource (http://morphosource.org) and DigiMorph (http://digimorph.org), and the majority of specimens (~83%), checked firsthand by us, are reposited at seven institutions, including Chengdu Institute of Biology (CIB), Chinese Academy of Sciences, Chengdu, Sichuan Province, China; Field Museum of Natural History (FMNH), Chicago, IL, USA; Department of Biology, Liupanshui Normal University, Liupanshui City, Guizhou Province, China; Zunyi Medical University, Zunyi City, Guizhou Province, China; Peking University of Paleontological Collection, Peking University, Beijing, China; Research Center of Palaeontology and Stratigraphy, College of Earth Sciences, Jilin University, Changchun, Jilin Province, China; and Zhejiang Museum of Natural History, Hangzhou, Zhejiang Province, China (data S1).

The specimens that we scanned used one of three micro–computed tomography (micro-CT) scanners, including the Quantum GX Micro-CT Imaging System (PerkinElmer, Waltham, USA) at CIB; the GE Phoenix v/tome/x 240-kv/180-kv scanner (Boston, USA) at the PaleoCT Laboratory in University of Chicago, Chicago, IL, USA; or the Nikon XT H 320 LC CT scanner at the Industrial Micro-CT laboratory at China University of Geosciences, Beijing, China. A detailed list of parameters for CT scans conducted by us, and for the datasets that we accessed from online platforms, is available in data S1. Segmentation and rendering of CT data were processed in VG Studio Max (version 2.2; Volume Graphics, Heidelberg, Germany), with images illustrated and assembled in Adobe Photoshop CC (Adobe System Inc., San Jose, USA). To analyze the ossification sequences of the carpus and tarsus through ontogeny, the snout-pelvic length was measured from the snout tip to the posterior extremity of the pelvic girdle by VG Studio Max 2.2 and ImageJ (version 1.53f51).

### Mesopodium and anatomical terms

The first scheme to divide the mesopodium was introduced by Gegenbaur (*2*, *73*), who proposed three transverse rows based on the spatial relationships with the zeugopodium and metapodium: the proximal row (radiale/tibiale, intermedium, and ulnare/fibulare), central row (element y, centralia, and element m), and the distal row (basale commune, distal carpals/tarsals, prehallux, and postminimus). However, the prevailing scheme of the mesopodium, created by Goette (*74*), is to divide it along the proximodistal axis of the limb into three longitudinal and paralleling columns: the preaxial column (radiale/tibiale and element y), central column (intermedium, centralia, and element m), and postaxial column (ulnare/fibulare) (*3*, *14*). The digital arch was coined by Schmalhausen (*15*) to represent series of branching and segmentation events from the basale commune that gives rise to metapodials and distal carpals/tarsals and was later refined [(*14*), pp. 339 and 361] to include the basale commune and distal carpals/tarsals in the mesopodium. The digital arch remains independent from the longitudinal columns in most taxa (*14*, *50*) but occasionally joins the postaxial column in certain species, in which the posterior distal tarsals and/or postminimus segment from the ulnare/fibulare [e.g., *T. marmoratus* in (*48*), *S. keyserlingii* in (*18*), and *Dicamptodon ensatus* in (*17*, *42*)]. Goette’s scheme patterned with the refined concept of digital arch is commonly used to date because it matches well with the early skeletogenesis of the limb (*14*): The radius/tibia distally segments to form radiale/tibiale, element y, and, if any, the prehallux; the ulna/fibula distally bifurcates to form the intermedium in the central column and the ulnare/fibulare in the postaxial column. In the central column, the centrale either segments from the intermedium or condense independently. The basale commune is a de novo condensation and segments into the distal carpals/tarsals and, if any, the postminimus to form the digital arch.

Here, we follow both schemes because we found that it is efficient for morphological descriptions by Gegenbaur’s scheme and it is coherent when referring developmental patterns by Goette’s scheme. Many anatomical terms have been introduced for the mesopodium over the past century, and we herein follow Shubin and Alberch (*14*) and Borkhvardt (*75*): element y [or centrale 1 in (*3*); mediale 1 in (*54*)], centrale close to intermedium as centrale 1 [or central proximal in (*3*)], centrale close to basale commune as centrale 2 [or centrale distal in (*3*)], and basale commune [or carpale/tarsale commune in (*3*)]. We also follow previous studies to use digits to include both the metapodium and phalanges [e.g., (*21*, *42*)].

## Supplementary Materials

### This PDF file includes:

Figs. S1 to S5 References

## Notes

View/request a protocol for this paper from *Bio-protocol*.
