# Supplementary material for: Ossification patterns of the carpus and tarsus in salamanders and impacts of preaxial dominance on the fin-to-limb transition
Source: Sci Adv. 2022 Oct 14;8(41):eabq7669. doi: 10.1126/sciadv.abq7669 (PMC9565805; doi:10.1126/sciadv.abq7669)
Supplement: Supplementary file 1 — Figs. S1 to S5 References [file sciadv.abq7669_sm.pdf]

Science Advances
AAAS

# Supplementary Materials for

## Ossification patterns of the carpus and tarsus in salamanders and impacts of preaxial dominance on the fin-to-limb transition

Jia Jia *et al.*

Corresponding author: Jia Jia, jia.jia@ucalgary.ca; Neil H. Shubin, nshubin@uchicago.edu;
Ke-Qin Gao, kqgao@pku.edu.cn

*Sci. Adv.* **8**, eabq7669 (2022)
DOI: 10.1126/sciadv.abq7669

**The PDF file includes:**

Figs. S1 to S5
Legends for data S1 and S2
References

**Other Supplementary Material for this manuscript includes the following:**

Data S1 and S2

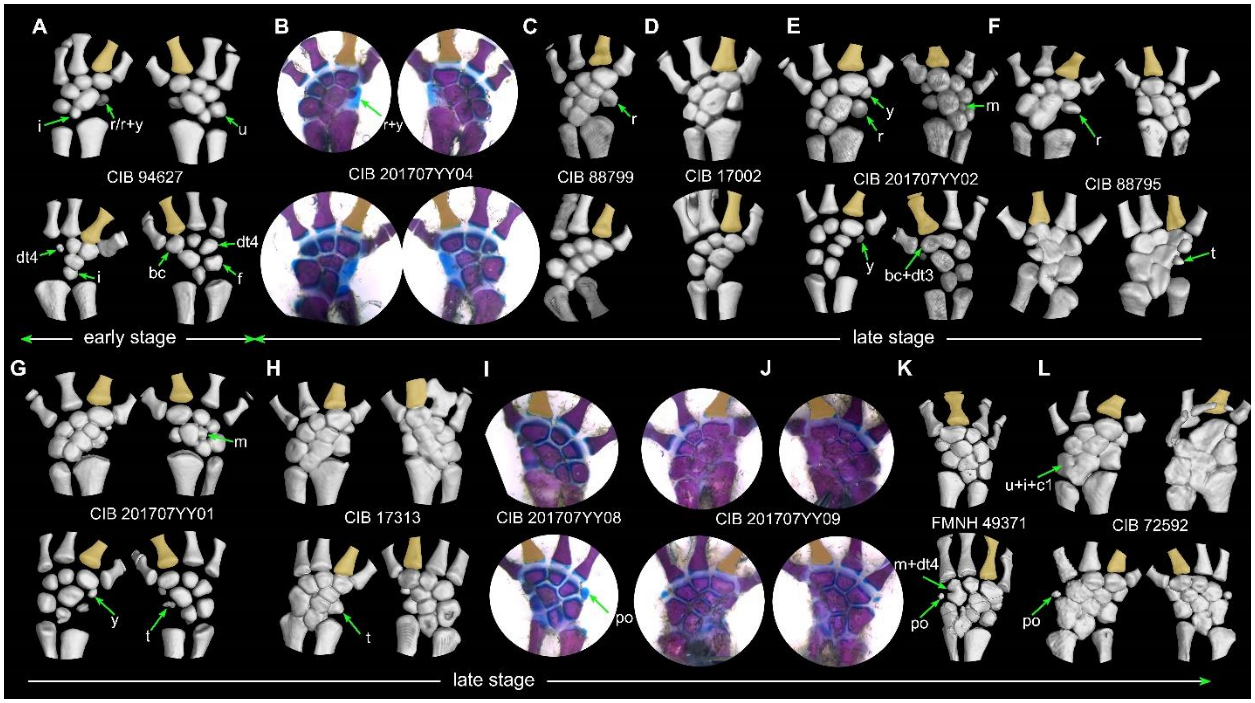


**Fig. S1.**

**Morphology and ossification patterns of the carpals and tarsals in extant four-toed hynobiid *Batrachuperus yenyuanensis*.** The manus (upper row) and pes (lower row) are arranged from left to right to show an increased ossification of the mesopodium (not to scale), and digit II is colored in gold to delineate the preaxial side. Most specimens (catalogue numbers located between carpus and tarsus) are shown in dorsal view except tarsus in CIB 88795 (**F**) and right-side carpus in CIB 72592 (**L**) are shown in ventral view to better visualize the mesopodials. Preaxial and postaxial dominance is present in the ossification of digital arch and non-digital-arch mesopodials, respectively. Proximal mesopodials ossify later than distal mesopodials in most specimens except the tibiale ossifies earlier than element y in CIB 17313 (**H**). Note the presence of an independent element m in the carpus (**E**, **G**) and an unfinished fusion (**K**) between element m and distal tarsal 4. Three specimens were cleared and double stained with cartilages stained in blue and bones in red. Carpus ossifies earlier than tarsus. See Fig. 2 for anatomical abbreviations and Data S1 for specimen details.

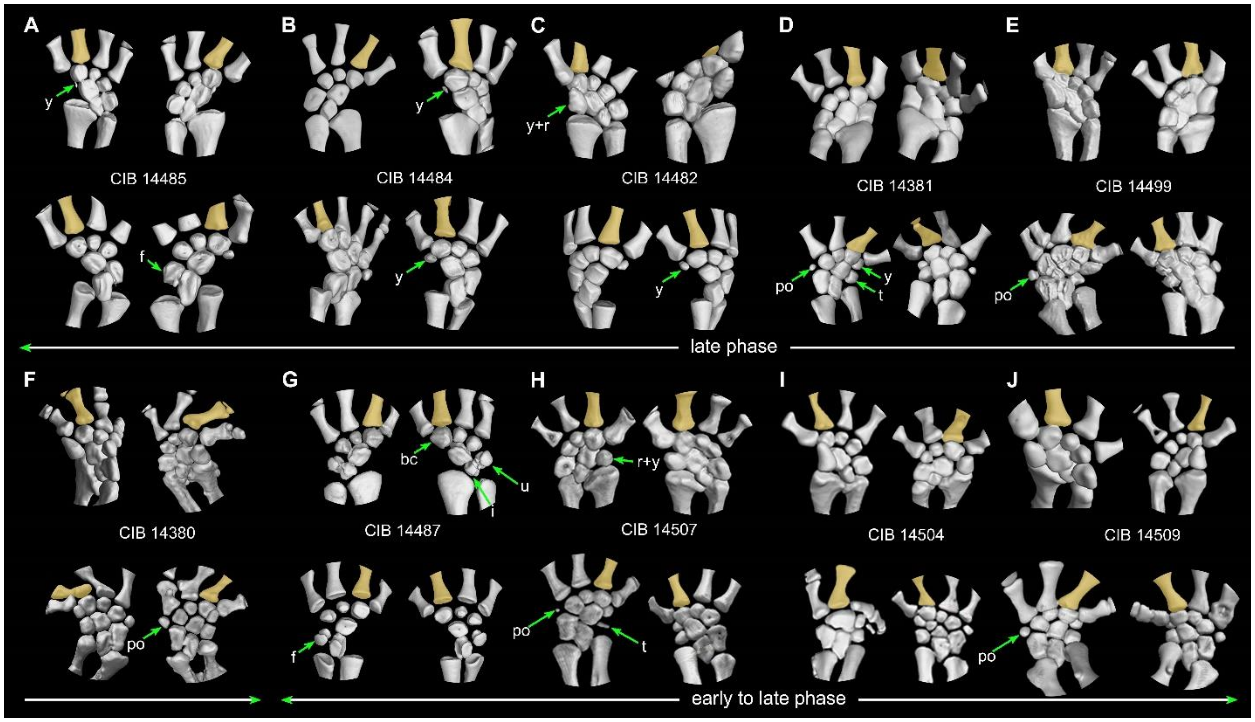


**Fig. S2.**

**Morphology and ossification patterns of the carpals and tarsals in neotenic (A–F) and metamorphosed (G–J) four-toed hynobiid *Batrachuperus londongensis*.** The manus (upper row) and pes (lower row) are arranged from left to right to show an increased ossification of the mesopodium (not to scale), with digit II colored in gold. Most specimens (catalogue numbers located between carpus and tarsus) are shown in dorsal view except carpus and tarsus in CIB 14485 (**A**) and CIB 14380 (**F**), left tarsus in CIB 14484 (**B**), and carpus of CIB 14482 (**C**), CIB 14499 (**E**), CIB 14507 (**H**), CIB 14504 (**I**), and CIB 14509 (**J**) are shown in ventral view to better visualize the mesopodials. The postminimus is the last to ossify in both neotenic and metamorphosed specimens. Ossification of non-digital-arch mesopodials is characterized by postaxial dominance. The relative weaker ossification of distal carpals/tarsals than basale commune in CIB 14487 (**G**) shows the presence of preaxial dominance in the ossification of the digital arch mesopodium. Carpus ossifies earlier than tarsus. See Fig. 2 for anatomical abbreviations and Data S1 for specimen details.

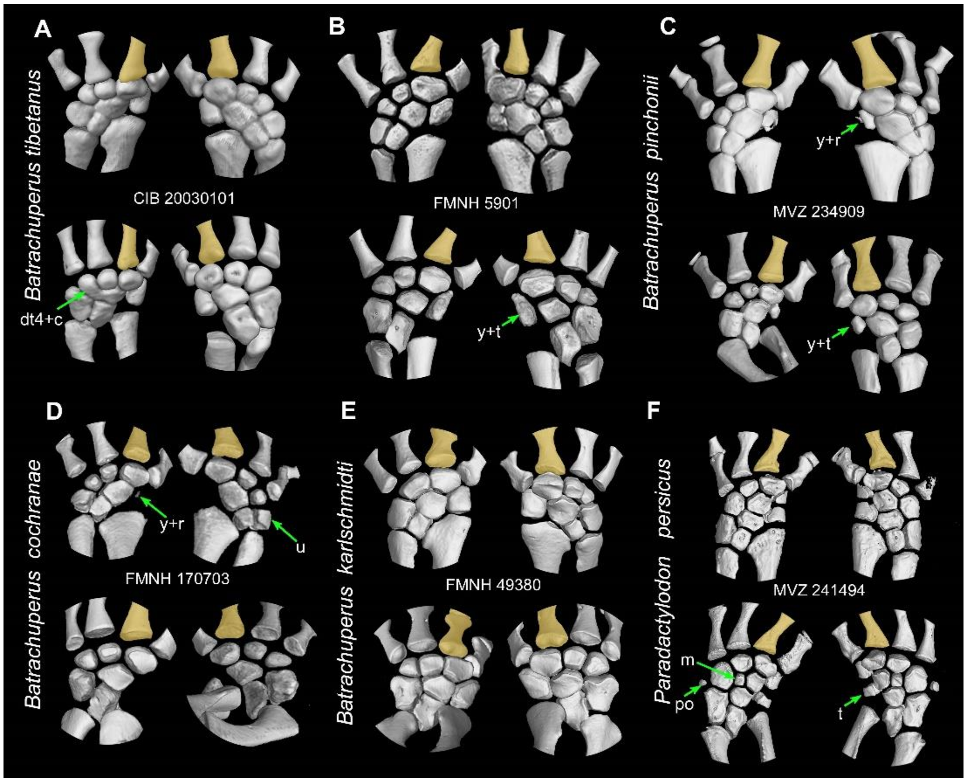


**Fig. S3.**

**Morphology and ossification patterns of the carpals and tarsals in five species of the four-toed hynobiid genera *Batrachuperus* and *Paradactylodon*.** (**A**, **B**) *Batrachuperus tibetanus*; (**C**) *Batrachuperus pinchonii*; (**D**) *Batrachuperus cochranae*; (**E**) *Batrachuperus karlschmidti*; (**F**) *Paradactylodon persicus*. Digit II is colored in gold to represent the preaxial side. The manus (upper row) and pes (lower row) in most specimens have a weak ossification in the preaxial column and demonstrate the presence of postaxial dominance in non-digital-arch mesopodium. The supernumerary element m is present in the tarsus of *Paradactylodon persicus* (**F**). All specimens are shown in dorsal view. Not to scale. See Fig. 2 for anatomical abbreviations and Data S1 for specimen details.

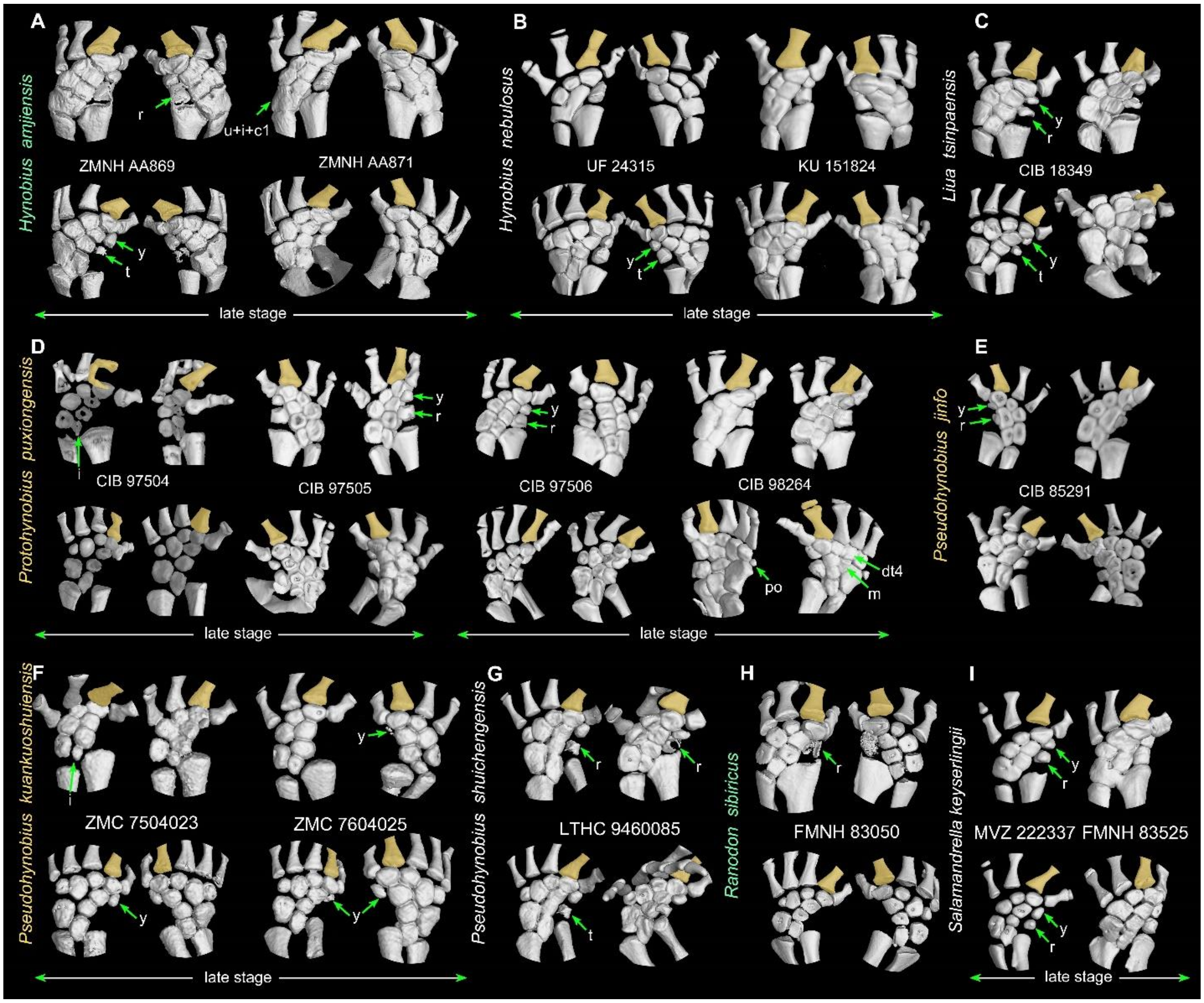


**Fig. S4.**

**Morphology and ossification patterns of the carpals and tarsals in nine extant species of Panhynobia.** Manus (upper row) and pes (lower row) are arranged from left to right for each species to reflect increased ossification of the mesopodium, and digit II is colored in gold to delineate the preaxial side. Most specimens (catalogue numbers located between carpus and tarsus) are shown in dorsal view except certain carpus and tarsus in CIB 18349 (**C**), CIB 97504-97506 (**D**), CIB 98264 (**D**), CIB 85291 (**E**), ZMC 7504023 (**F**), LTHC 9460085 (**G**) are shown in ventral view to better visualize the mesopodials. Postaxial dominance in non-digital-arch mesopodium are reflected by the weak ossifications of radiale/tibiale and element y in every species, and a distal-to-proximal ossification polarity is characterized by the earlier appearance or higher ossification extent of element y than radiale/tibiale in the preaxial column (**A**, **C**, **F**, **G**) and the weak ossification extent of intermedium in the central column (**D**, **F**). An independent element m is present in the tarsus of CIB 98264 of *Protohynobius puxiongensis* (**D**), in which distal tarsal 4 lacks a proximal extension into the central row of the tarsals. Species with names colored in green have carpus ossify earlier than tarsus, that in yellow have carpus ossify later than tarsus and that in white remains indistinguishable. Not to scale. See Fig. 2 for anatomical abbreviations and Data S1 for specimen details.

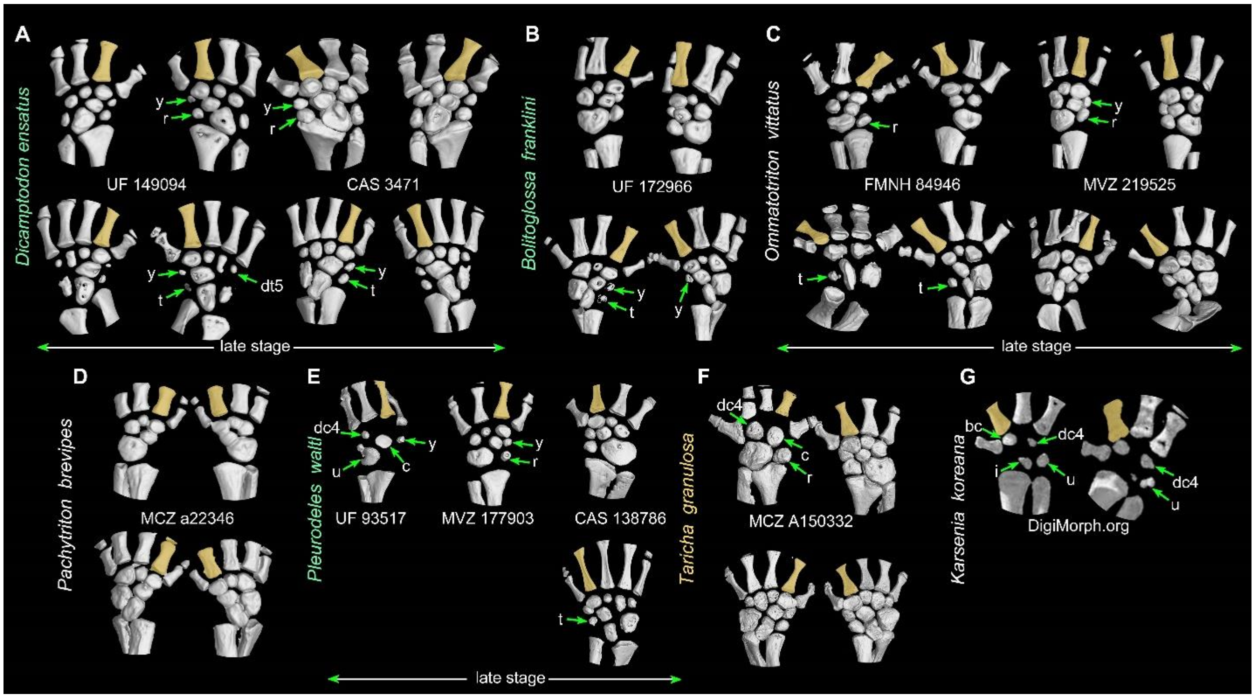


**Fig. S5.**

**Morphology and ossification patterns of the carpals (upper row) and tarsals (lower row) in seven extant species in Dicamptodontidae (A), Plethodontidae (B, G), and Salamandridae (C–F).** Digit II is colored in gold to represent the preaxial side of the limb. Most specimens (catalogue numbers located between carpus and tarsus) are shown in dorsal view except the left- and right-side carpus in CAS 3471 (**A**) and the left tarsus in FMNH 84946 (**C**) are shown in ventral view to better visualize the mesopodials. Postaxial dominance is present in the ossification of non-digital-arch mesopodials of all species, where element y and radiale/tibiale are weakly ossified or absent; and also exists in the ossification of digital arch mesopodium of *Bolitoglossa* (**B**), *Pleurodeles* (**E**) and *Taricha* (**F**) and *Karsenia* (**G**), in which distal carpal/tarsal 4 is the first to ossify (**E**, **F**) or has ossification extents higher than (**B**) or similar to (**G**) that of basale commune. Ossifications in the preaxial (**C**, **F**) and central (**E**, **G**) columns proceed from proximally to distally, and is reversed in the preaxial column of *Bolitoglossa* (**B**) and *Pleurodeles* (**E**). Species with names colored in green have carpus ossify earlier than tarsus, that in yellow have carpus ossify later than tarsus and that in white remains indistinguishable. Not to scale. See Fig. 2 for anatomical abbreviations and Data S1 for specimen details.

**Data S1. (separate file)**

Snout-pelvic length, life history strategy, ossification patterns of the mesopodium and CT scan parameters for specimens investigated in this study.

**Data S2. (separate file)**

Species investigated in this study and their taxonomy, life history strategy, larval type and ecological preference at adult stage out of breeding season.

## REFERENCES AND NOTES


1. R. Owen, *On the Nature of Limbs* (Van Voorst, 1843).

2. C. Gegenbaur, *Untersuchungen zur Vergleichenden Anatomie der Wirbelthiere. Volume I. Carpus und Tarsus* (Verlag Von Wilhelm Engelmann, 1864).

3. N. Holmgren, On the origin of the tetrapod limb. *Acta Zoologica* **14**, 185–295 (1933).

4. B. K. Hall, *Fins into Limbs: Evolution, Development, and Transformation* (The University of Chicago Press, 2007).

5. J. Zakany, D. Duboule, The role of *Hox* genes during vertebrate limb development. *Curr. Opin. Genet. Dev.* **17**, 359–366 (2007).

6. J. M. Woltering, I. Irisarri, R. Ericsson, J. M. P. Joss, P. Sordino, A. Meyer, Sarcopterygian fin ontogeny elucidates the origin of hands with digits. *Sci. Adv.* **6**, eabc3510 (2020).

7. M. I. Coates, J. E. Jeffery, M. Ruta, Fins to limbs: What the fossils say. *Evol. Dev.* **4**, 390–401 (2002).

8. C. A. Boisvert, E. Mark-Kurik, P. E. Ahlberg, The pectoral fin of *Panderichthys* and the origin of digits. *Nature* **456**, 636–638 (2008).

9. N. H. Shubin, E. B. Daeschler, F. A. Jenkins Jr., The pectoral fin of *Tiktaalik roseae* and the origin of the tetrapod limb. *Nature* **440**, 764–771 (2006).

10. R. Cloutier, A. M. Clement, M. S. Y. Lee, R. Noël, I. Béchard, V. Roy, J. A. Long, *Elpistostege* and the origin of the vertebrate hand. *Nature* **579**, 549–554 (2020).

11. Z. Johanson, J. Joss, C. A. Boisvert, R. Ericsson, M. Sutija, P. E. Ahlberg, Fish fingers: Digit homologues in sarcopterygian fish fins. *J. Exp. Zool.* **308B**, 757–768 (2007).

12. A. R. Gehrke, I. Schneider, E. Calle-Mustienes, J. J. Tena, C. Gomez-Marin, M. Chandran, T. Nakamura, I. Braasch, J. H. Postlethwait, J. L. Gómez-Skarmeta, N. Shubin, Deep conservation of wrist and digit enhancers in fish. *Proc. Natl. Acad. Sci. U.S.A.* **112**, 803–808 (2014).

13. T. Nakamura, A. R. Gehrke, J. Lemberg, J. Szymaszek, N. H. Shubin, Digits and fin rays share common developmental histories. *Nature* **537**, 225–228 (2016).

14. N. H. Shubin, P. Alberch, A morphogenetic approach to the origin and basic organization of the tetrapod limb. *Evol. Biol.* **20**, 319–387 (1986).

15. I. I. Schmalhausen, Die Entwickelung des Extremitätenskelettes von *Salamandrella keyserlingii*. *Anat. Anz.* **37**, 431–446 (1910).

16. N. H. Shubin, The implications of "The Bauplan" for development and evolution of the tetrapod limb, in *Developmental Patterning of the Vertebrate Limb*, J. R. Hinchliffe, J. M. Hurle, D. Summerbell, Eds. (Plenum, 1991), pp. 411–421.

17. N. H. Shubin, D. B. Wake, Morphological variation, development, and evolution of the limb skeleton of salamanders, in *Amphibia Biology, Volume 5: Osteology*, H. Heatwole, M. Davies, Eds. (Surrey Beatty and Sons, 2003), pp. 1782–1808.

18. E. I. Vorobyeva, J. R. Hinchliffe, Developmental pattern and morphology of *Salamandrella keyserlingii* limbs (Amphibia, Hynobiidae) including some evolutionary aspects. *Russ. J. Herpetol.* **3**, 68–81 (1996).

19. J. R. Hinchliffe, P. Tidball, T. J. Staughton, S. Wade, E. I. Vorobyeva, New immunohistochemical methods and their use for evolutionary-developmental analysis of heterochronic transformation of the patterns of limb skeletal development in tailed amphibians, in *Current Studies in Herpetology*, C. Miaud, R. Guyétant, Eds. (SHE, 1998), pp. 197–206.

20. S. B. Marks, “Development and evolution of the Dusky Salamanders (genus *Desmognathus*),” thesis, University of California, Berkeley, CA (1995).

21. R. A. Franssen, S. Marks, D. Wake, N. H. Shubin, Limb chondrogenesis of the seepage salamander, *Desmognathus aeneus* (Amphibia: Plethodontidae). *J. Morphol.* **265**, 87–101 (2005).

22. R. R. Kerney, J. Hanken, D. C. Blackburn, Early limb patterning in the direct-developing salamander Plethodon cinereus revealed by *sox9* and *col2a1*. *Evol. Dev.* **20**, 100–107 (2018).

23. I. I. Schmalhausen, On the extremeties of *Ranidens sibiricus* Kessl. *Zool. Zhurnal* **2**, 129–135 (1917).

24. E. I. Vorobyeva, O. P. Olʼshevskaya, R. J. Hinchliffe, Specific features of development of the paired limbs in *Ranodon sibiricus* Kessler (Hynobiidae, Caudata). *Ontogenez* **28**, 188–197 (1997).

25. J. R. Hinchliffe, E. I. Vorobyeva, Developmental basis of limb homology in urodeles: heterochronic evidence from the primitive hynobiid family, in *Homology*, G. R. Bock, G. Cardew, Eds. (JWS, 1999), pp. 95–109.

26. K. Erdmann, Zur Entwicklung des knöchernen Skelets von *Triton* und *Rana* unter besonderer Berücksichtigung der Zeitfolge der Ossifikationen. *Z. Anat. Entwicklungs.* **101**, 566–651 (1933).

27. O. Rieppel, Die Gliedmassen der Tetrapoden – ein aktuelles problem der evolutionsforschung. *Sci. Nat.* **80**, 295–301 (1993a).

28. N. B. Fröbisch, Ossification patterns in the tetrapod limb—Conservation and divergence from morphogenetic events. *Biol. Rev.* **83**, 571–600 (2008).

29. J. C. Olori, Skeletal morphogenesis of *Microbrachis* and *Hyloplesion* (Tetrapoda: Lepospondyli), and implications for the developmental patterns of extinct, early tetrapods. *PLOS ONE* **10**, e0128333 (2015).

30. J. Jia, K.-Q. Gao, A new hynobiid-like salamander (Amphibia, Urodela) from Inner Mongolia, China, provides a rare case study of developmental features in an Early Cretaceous fossil urodele. *PeerJ* **4**, e2499 (2016).

31. N. B. Fröbisch, N. H. Shubin, Salamander limb development: Integrating genes, morphology and fossils. *Dev. Dyn.* **240**, 1087–1099 (2011).

32. N. B. Fröbisch, R. L. Carroll, R. R. Schoch, Limb ossification in the Paleozoic branchiosaurid *Apateon* (Temnospondyli) and the early evolution of preaxial dominance in tetrapod limb development. *Evol. Dev.* **9**, 69–75 (2007).

33. N. B. Fröbisch, C. Bickelmann, J. C. Olori, F. Witzmann, Deep-time evolution of regeneration and preaxial polarity in tetrapod limb development. *Nature* **527**, 231–234 (2015).

34. A. Trofka, B.-L. Huang, J. Zhu, W. F. Heinz, V. Magidson, Y. Shitaba, Y.-B. Shi, B. Tarchini, H. S. Stadler, M. Kabangu, N. W. Ai Haj Baddar, S. R. Voss, S. Mackem, Genetic basis for an evolutionary shift from ancestral preaxial to postaxial limb polarity in non-urodele vertebrates. *Curr. Biol.* **31**, 4923–4934.e5 (2021).

35. J. S. Anderson, R. R. Reisz, D. Scott, N. B. Fröbisch, S. S. Sumida, A stem batrachian from the Early Permian of Texas and the origin of frogs and salamanders. *Nature* **453**, 515–518 (2008).

36. D. Dilkes, Carpus and tarsus of Temnospondyli. *Vertebr. Anat. Morphol. Palaeontol.* **1**, 51–87 (2015).

37. Z. Roček, L. Dong, M. Fabrezi, Y. Rong, Y. Wang, Carpus in Mesozoic anurans: The Early Cretaceous anuran *Genibatrachus* from northeastern China. *Cretac. Res.* **129**, 104984 (2022).

38. J. Jia, J. S. Anderson, K.-Q. Gao, New material of the stem hynobiid *Neimengtriton daohugouensis* comb. nov. (Wang, 2004) from the Middle Jurassic of China yields insights into variation and evolutionary patterns of limb and vertebra in early salamanders (Amphibia, Urodela). *iScience* **24**, 102744 (2021).

39. J. Jia, K.-Q. Gao, A new stem hynobiid salamander (Urodela, Cryptobranchoidea) from the Upper Jurassic (Oxfordian) of Liaoning Province, China. *J. Vertebr. Paleontol.* **39**, e1588285–22 (2019).

40. H. L. D. Nye, J. A. Cameron, E. A. G. Chernoff, D. L. Stocum, Extending the table of stages of normal development of the axolotl: Limb development. *Dev. Dyn.* **226**, 555–560 (2003).

41. E. L. Bierbaum, "Understanding the homology and variation of mesopodial elements and digit number in the extremely limb reduced salamander family Amphiumidae," thesis, Southeastern Louisiana University, Hammond, LA (2019).

42. D. B. Wake, N. H. Shubin, Limb development in the Pacific giant salamanders, *Dicamptodon* (Amphibia, Caudata, Dicamptodontidae). *Can. J. Zool.* **76**, 2058–2066 (1998).

43. V. G. Borkhvardt, A. A. Moisseeva, A. V. Ledentsov, Development of mesopodial skeleton of Siberian Salamander, *Salamandrella keyserlingii* (Amphibia, Hynobiidae), at postmetamorphous stages of ontogenesis. *Zool. Zhurnal* **71**, 64–73 (1992).

44. K. Iizuka, N. A. Poyarkov, H. Koishi, Y. Shiraiwa, S. K. Sessions, A. Vassilieva, Limb development of Japanese clawed salamander, *Onychodactylus japonicus* (Houttuyn 1782). *Bull. Biogeogr. Soc. Jpn.* **65**, 13–19 (2010).

45. N. H. Shubin, D. B. Wake, Implications of direct development for the tetrapod limb bauplan. *Am. Zool.* **31**, 8A (1991).

46. D. B. Wake, J. Hanken, Direct development in the lungless salamanders: What are the consequences for developmental biology, evolution and phylogenesis? *Int. J. Dev. Biol.* **40**, 859–869 (1996).

47. S. B. Marks, A. Collazo, Direct development in *Desmognathus aeneus* (Caudata: Plethodontidae): A staging table. *Copeia* **1998**, 637–648 (1998).

48. A. N. Sewertzoff, Studien über die Entwicklung der Muskeln, Nerven und des Skeletts der Extremitäten der niederen Tetrapoda. *Bull. Soc. Imp. Nat. Mosc.* **21**, 1–430 (1908).

49. M. J. Blanco, P. Alberch, Caenogenesis, developmental variability, and evolution in the carpus and tarsus of the marbled newt *Triturus marmoratus*. *Evolution* **46**, 677–687 (1992).

50. N. H. Shubin, D. B. Wake, A. J. Crawford, Morphological variation in the limbs of *Taricha granulosa* (Caudata: Salamandridae): Evolutionary and phylogenetic implications. *Evolution* **49**, 874–884 (1995).

51. E. Jarvik, On the structure of the snout of crossopterygians and lower gnathostomes in general. *Zoologische Beitrage Aus Uppsala* **21**, 235–675 (1942).

52. E. Jarvik, On the origin of girdles and paired fins. *Isr. J. Zool.* **14**, 141–172 (1965).

53. G. P. Wagner, P. A. Khan, M. J. Blanco, B. Misof, R. A. Liversgae, Evolution of *Hoxa-11* expression in amphibians: Is the urodele autopodium an innovation? *Am. Zool.* **39**, 686–694 (1999).

54. I. I. Schmalhausen, *Development of the Extremities of the Amphibia and their Significance to the Question of the Origins of the Vertebrates* (University Press, 1915).

55. J. R. Hinchliffe, E. I. Vorobyeva, J. Géraudie, Is there a tetrapod developmental bauplan underlying limb evolution? Evidence from a teleost fish and from urodele and anuran amphibians. *Syst. Assoc. Spec. Vol.* **61**, 377–391 (2001).

56. O. Rieppel, Studies on skeleton formation in reptiles: Patterns of ossification in the skeleton of *Chelydra serpentina* (Reptilia, Testudines). *J. Zool.* **231**, 487–509 (1993b).

57. H. Iwasawa, Y. Kera, Normal stages of development of the Japanese lungless salamander, *Onychodactylus japonicus* (Houttuyn). *Japan. J. Herpetol.* **8**, 73–89 (1980).

58. H. Iwasawa, K. Yamashita, Normal stages of development of a hynobiid salamander, *Hynobius nigrescens* Stejneger. *Curr. Herpetol.* **8**, 73–89 (1991).

59. M. R. Sánchez-Villagra, Comparative patterns of postcranial ontogeny in therian mammals: An analysis of relative timing of ossification events. *J. Exp. Zool.* **294**, 264–273 (2002).

60. V. G. Borkhvardt, Comparative study of the development of limbs in larvae of the common frog *Rana temporaria* (Amphibia: Anura) and in *Salamandrella* (Caudata). *Russ. J. Herpetol.* **3**, 58–67 (1996).

61. F. Witzmann, Developmental patterns and ossification sequence in the Permo-Carboniferous temnospondyl *Archegosaurus decheni* (Saar-Nahe Basin, Germany). *J. Vertebr. Paleontol.* **26**, 7–17 (2006).

62. M. Coates, New palaeontological contributions to limb ontogeny and phylogeny, in *Developmental Patterning of the Vertebrate Limb*, J. R. Hinchliffe, J. M. Hurle, D. Summerbell, Eds. (Plenum, 1991), pp. 325–337.

63. A. R. Milner, S. E. K. Sequeira, The temnospondyl amphibians from the Viséan of East Kirkton, West Lothian, Scotland. *Trans. R. Soc. Edinb. Earth Sci.* **84**, 331–361 (1994).

64. S. J. Godfrey, The postcranial skeletal anatomy of the Carboniferous tetrapod *Greererpeton burkemorani*. *Philos. Trans. R. Soc. Lond. B, Biol. Sci.* **323**, 75–133 (1989).

65. R. B. Holmes, The Carboniferous amphibian *Proterogyrinus scheelei* Romer, and the early evolution of tetrapods. *Philos. Trans. R. Soc. Lond. B, Biol. Sci.* **306**, 431–527 (1984).

66. J. A. Clack, C. E. Bennett, D. K. Carpenter, S. J. Davies, N. C. Fraser, T. I. Kearsey, J. E. A. Marshall, D. Millward, B. K. A. Otoo, E. J. Reeves, A. J. Ross, M. Ruta, K. Z. Smithson, T. R. Smithson, S. A. Walsh, Phylogenetic and environmental context of a Tournaisian tetrapod fauna. *Nat. Ecol. Evol.* **1**, 0002 (2016).

67. M. R. Whitney, S. E. Pierce, Osteohistology of *Greererpeton* provides insight into the life history of an early Carboniferous tetrapod. *J. Anat.* **239**, 1256–1272 (2021).

68. B. K. A. Otoo, J. R. Bolt, R. E. Lombard, K. D. Angielczyk, M. I. Coates, The postcranial anatomy of *Whatcheeria deltae* and its implications for the family Whatcheeriidae. *Zool. J. Linn. Soc.* **193**, 700–745 (2021).

69. A. Warren, S. Turner, The first stem tetrapod from the Lower Carboniferous of Gondwana. *Palaeontology* **47**, 151–184 (2004).

70. O. A. Lebedev, M. I. Coates, The postcranial skeleton of the Devonian tetrapod *Tulerpeton curtum* Lebedev. *Zool. J. Linn. Soc.* **114**, 307–348 (1995).

71. G. F. Stopper, G. P. Wagner, Inhibition of sonic hedgehog signaling leads to posterior digit loss in *Ambystoma mexicanum*: Parallels to natural digit reduction in urodeles. *Dev. Dyn.* **236**, 321–331 (2007).

72. N. H. Shubin, D. B. Wake, Phytogeny, variation, and morphological integration. *Am. Zool.* **36**, 51–60 (1996).

73. C. Gegenbaur, *Vergleichende Anatomie der Wirbelthiere: mit Berücksichtigung der Wirbellosen* (Verlag Von Wilhelm Engelmann, 1898).

74. A. Goette, *Üeber Entwickelung and regeneration des Gliedmassenskelets der Molche* (Verlag Von Leopold Voss, 1879).

75. V. G. Borkhvardt, Development of the limbs in larvae of Siberian Salamander, *Salamandrella keyserlingii* (Amphibia, Hynobiidae). *Zool. Zhurnal* **73**, 53–67 (1994).

76. M. Y. Chen, R. L. Mao, D. Liang, M. Kuro-o, X. M. Zeng, P. Zhang, A reinvestigation of phylogeny and divergence times of Hynobiidae (Amphibia, Caudata) based on 29 nuclear genes. *Mol. Phylogenet. Evol.* **83**, 1–6 (2015).

77. R. A. Nussbaum, Geographic variation and systematics of salamanders of the genus *Dicamptodon* Strauch (Ambystomatidae). *Misc. Publ. Mus. Zool. Univ. Mich.* **149**, 1–94 (1976).

78. K.-Q. Gao, N. H. Shubin, Late Jurassic salamanders from northern China. *Nature* **410**, 574–577 (2001).

79. Z. Dong, Y. Wang, A new urodele (*Liaoxitriton zhongjiani* gen. et sp. nov.) from the Early Cretaceous of western Liaoning Province, China. *Vert. PalAsiat.* **36**, 159–172 (1998).

80. Y. Wang, A new Mesozoic caudate (*Liaoxitriton daohugouensis* sp. nov.) from Inner Mongolia. *Sci. Bull.* **49**, 858–860 (2004).

81. Y. F. Rong, Restudy of *Regalerpeton weichangensis* (Amphibia: Urodela) from the Lower Cretaceous of Hebei, China. *Vert. PalAsiat.* **56**, 121–136 (2018).

82. D. A. Good, D. B. Wake, Geographic variation and speciation in the Torrent Salamanders of the genus *Rhyacotriton* (Caudata: Rhyacotritonidae). *Univ. Calif. Publ. Zool.* **126**, 1–91 (1992).
